# Self-assembled copper-based nanoparticles for enzyme catalysis-enhanced chemodynamic/photodynamic/antiangiogenic tritherapy against hepatocellular carcinoma

**DOI:** 10.1186/s12951-024-02626-x

**Published:** 2024-06-26

**Authors:** Yaping Wang, Xun Zhang, Yunfeng Ma, Xiaobo Zhou, Weijun Xu, Sida Qin, Chengcheng Yang

**Affiliations:** 1 Department of Oncology, The First Affiliated Hospital of Xi’an Jiaotong University, Xi’an, 710061 China; 2 State Key Laboratory for Mechanical Behavior of Materials, Xi’an Jiaotong University, Xi’an, 710049 China; 3 Department of Thoracic Surgery, The First Affiliated Hospital of Xi’an Jiaotong University, Xi’an, 710061 China; 4 Department of Pathogenic Biology and Immunology, School of Basic Medical Sciences, Xi’an Jiaotong University, Xi’an, 710061 China

**Keywords:** Reactive oxygen species, Coordination self-assembly, Copper nanozyme, Antiangiogenesis, Synergistic therapy

## Abstract

As an emerging cancer treatment strategy, reactive oxygen species-based tumor catalytic therapies face enormous challenges due to hypoxia and the overexpression of glutathione (GSH) in the tumor microenvironment. Herein, a self-assembled copper-based nanoplatform, TCCHA, was designed for enzyme-like catalysis-enhanced chemodynamic/photodynamic/antiangiogenic tritherapy against hepatocellular carcinoma. TCCHA was fabricated from Cu^2+^, 3,3′-dithiobis (propionohydrazide), and photosensitizer chlorine e6 via a facile one-pot self-assembly strategy, after which an aldehyde hyaluronic acid was coated, followed by loading of the antivascular drug AL3818. The obtained TCCHA nanoparticles exhibited pH/GSH dual-responsive drug release behaviors and multienzymatic activities, including Fenton, glutathione peroxidase-, and catalase-like activities. TCCHA, a redox homeostasis disruptor, promotes ⋅OH generation and GSH depletion, thus increasing the efficacy of chemodynamic therapy. TCCHA, which has catalase-like activity, can also reinforce the efficacy of photodynamic therapy by amplifying O_2_ production. In vivo, TCCHA efficiently inhibited tumor angiogenesis and suppressed tumor growth without apparent systemic toxicity. Overall, this study presents a facile strategy for the preparation of multienzyme-like nanoparticles, and TCCHA nanoparticles display great potential for enzyme catalysis-enhanced chemodynamic/photodynamic/antiangiogenic triple therapy against cancer.

**Graphical Abstract:**

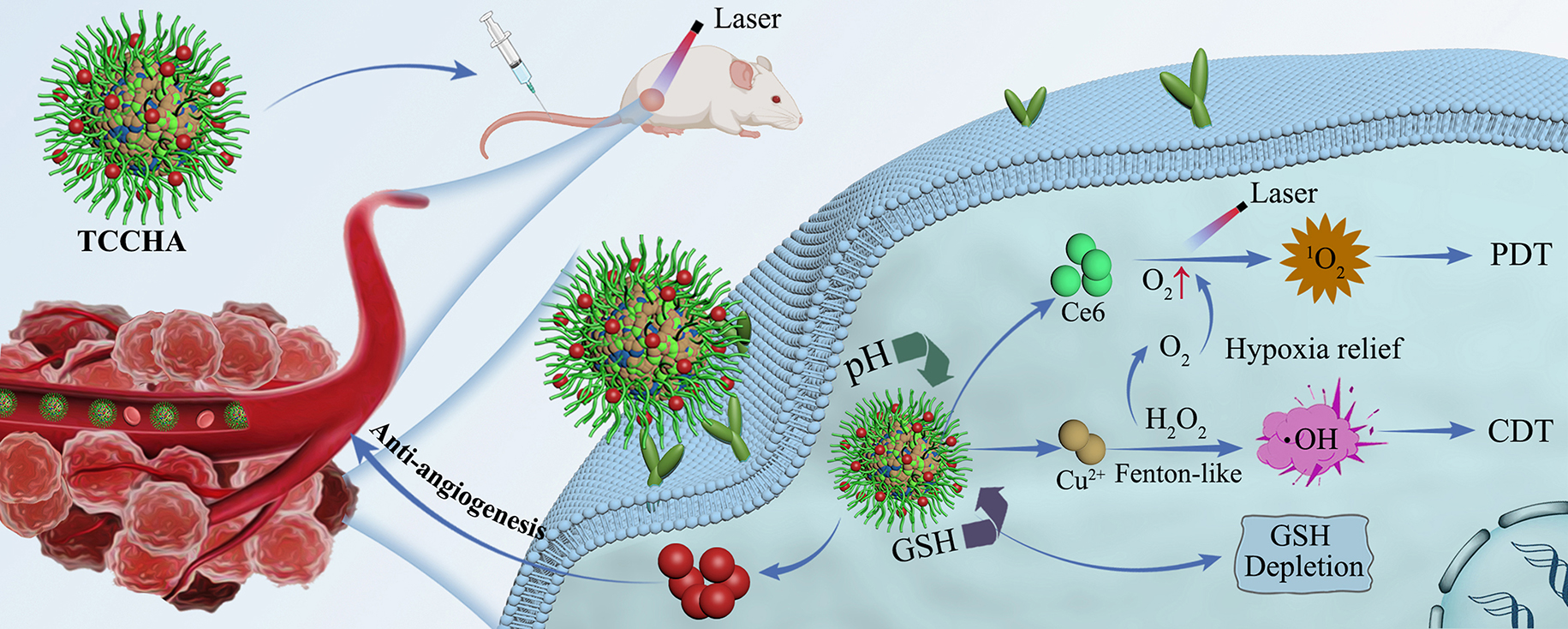

**Supplementary Information:**

The online version contains supplementary material available at 10.1186/s12951-024-02626-x.

## Introduction

Hepatocellular carcinoma, characterized by high metastatic potential and frequent recurrence, has become a major threat to human health worldwide. Despite remarkable progress in hepatocellular carcinoma clinical therapy, the need for advanced therapeutic strategies remains urgent [1–3]. Reactive oxygen species (ROS)-based therapeutics, such as photodynamic therapy (PDT), chemodynamic therapy (CDT), and sonodynamic therapy, have garnered increasing interest and hold great promise for hepatocellular carcinoma treatment [4–6]. In general, ROS, including oxygen-containing chemically reactive molecules, such as oxygen (^1^O_2_), hydroxyl radical (·OH), peroxide (O_2_^−^), and superoxide (·O_2_^−^), play critical roles in regulating the balance of cellular redox homeostasis [7, 8]. Excessive accumulation of intracellular ROS can trigger oxidative stress, disrupt cellular functions and lead to programmed cell death or apoptosis, which can have beneficial effects on tumor therapy [9, 10]. Photodynamic therapy (PDT) is a ROS-based therapeutic strategy that primarily relies on the generation of ^1^O_2_ by excited photosensitizers (PSs) in the presence of O_2_ and has been widely recognized for cancer treatment due to its specific spatiotemporal selectivity [11, 12]. As O_2_ serves as the source of ^1^O_2_ in PDT, the concentration of O_2_ directly impacts the level of ^1^O_2_ within the tumor. Unfortunately, the hypoxic microenvironment in tumors can limit the efficacy of PDT [13]. In addition, chemodynamic therapy (CDT) has recently emerged as another ROS-based therapeutic strategy that relies on Fenton and Fenton-like reactions and uses metal ions to catalyze the overexpression of endogenous H_2_O_2_ in tumors to generate highly toxic ⋅OH without the aid of external energy [14, 15]. Moreover, site-specific ROS production in CDT can effectively prevent severe systemic toxicity due to the low level of H_2_O_2_ in normal cells [16]. Furthermore, while specific metal ions in CDT catalyze H_2_O_2_ to produce ⋅OH, they can also stimulate the generation of O_2_ through the Haber–Weiss reaction, thereby alleviating tumor hypoxia [17]. Excitingly, the oxygen generation ability of special metal ions in CDT can be harnessed by PDT to enhance its therapeutic efficacy, thereby achieving synergistic therapeutic efficacy of CDT-PDT in combination therapy [18, 19]. However, the defense system in cells against ROS-induced oxidative stress, known as the intracellular antioxidant defense system, poses a major challenge to both PDT and CDT. In particular, the overexpression of the antioxidant glutathione (GSH) in tumor cells could scavenge ROS, thereby weakening the effects of PDT and CDT [20, 21]. Therefore, integrating CDT and PDT, along with the simultaneous elimination of GSH to restore ROS in tumor cells, is a highly sought-after new strategy that holds promising prospects for achieving more effective ROS-mediated therapies.

In recent years, nanozymes, which are artificial enzymes that mimic natural enzymatic activity, have garnered tremendous attention in cancer treatment [22]. Among these, there are inorganic nanozymes containing multivalent metal ions, such as Fe^2+^/Fe^3+^ and Cu^+^/Cu^2+,^ which possess metal ion redox couples that can exhibit glutathione peroxidase (GPx)-like, peroxidase (POD)-like, or catalase (CAT)-like activities [23]. These nanozymes have demonstrated remarkable efficacy in modulating the tumor microenvironment (TEM), resulting in enhanced therapeutic effects against tumors. Compared to Fe^2+^/Fe^3+^-mediated Fenton agents, Cu^+^/Cu^2+^ has dramatically higher Fenton-like catalytic activity [24, 25]. In addition to exhibiting superior Fenton-like activity, Cu^2+^-based nanoplatforms also exhibit GPx-like activity, consuming intracellular GSH while concurrently generating more catalytically active Cu^+^ [26]. In addition, Cu^2+^-based nanoplatforms also possess CAT-like activity and convert endogenous H_2_O_2_ into O_2_, thus alleviating the hypoxic tumor microenvironment and generating abundant superoxide anions and hydroxyl radicals for enhanced hepatocellular carcinoma therapy [2, 27]. Despite Cu^2+^ being an essential nutrient that plays a crucial role in various physiological processes and holds enormous potential as a safe and effective approach for tumor catalytic therapy, it can also stimulate tumor cells to secrete multiple angiogenic factors [28], thereby promoting tumor angiogenesis and facilitating metastasis. Therefore, combining PDT with copper-mediated Fenton-/GPx-/CAT-like catalytic activities and simultaneously inhibiting copper-induced tumor angiogenesis may maximize therapeutic efficacy through a synergistic effect. Some studies have shown that anlotinib hydrochloride (AL3818), a novel multi-targeted receptor tyrosine kinase inhibitor, not only inhibits angiogenesis in hepatocellular carcinoma but also inhibits tumor proliferation and survival [29–31]. However, its tumor-specific delivery is still a challenge.

**Scheme 1:**
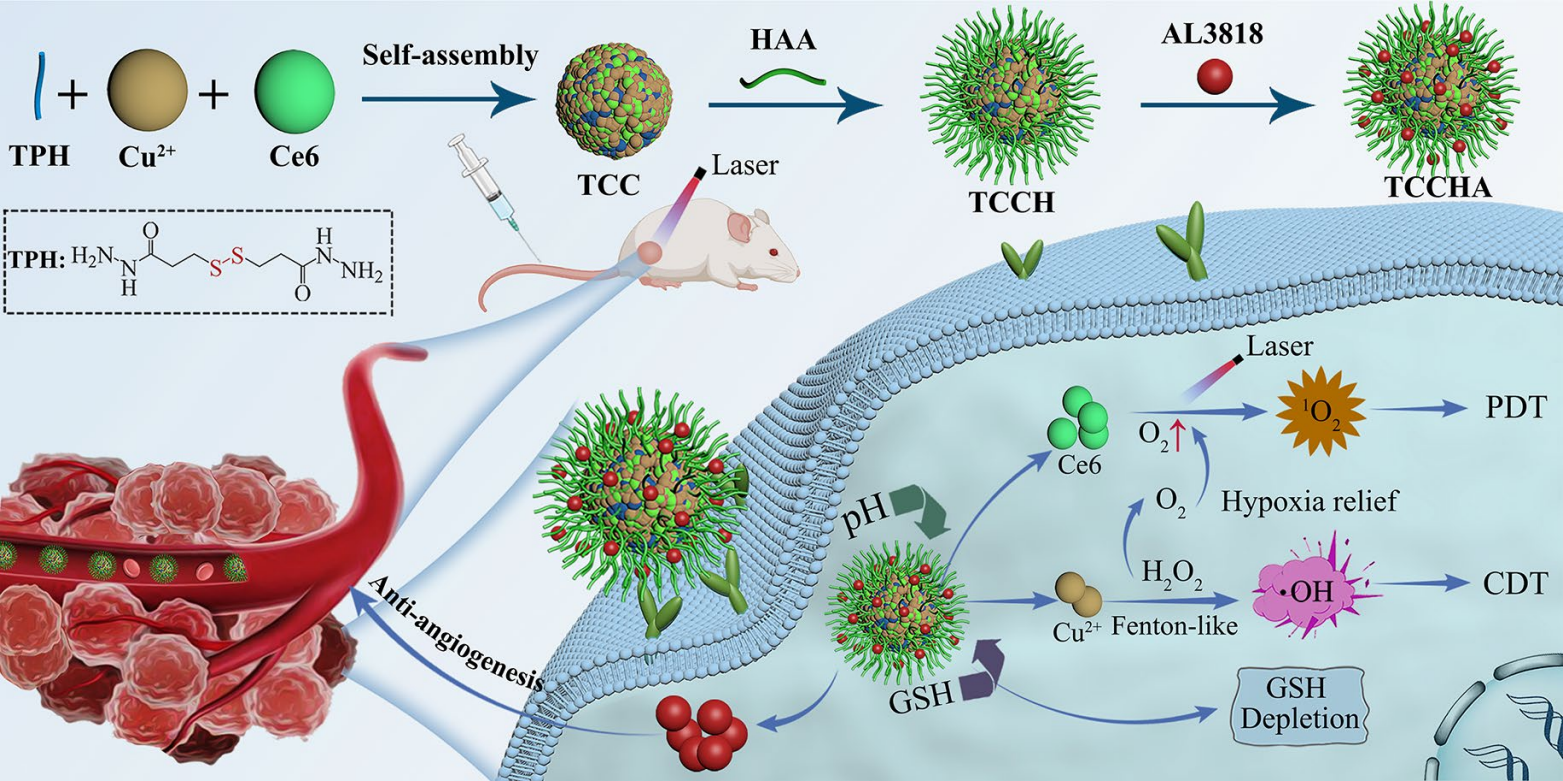
Schematic illustration of the fabrication process of TCCHA nanoparticles and their application as a ROS amplifier enhanced ROS-mediated CDT/PDT antitumor therapy

Herein, we propose a facile self-assembly strategy to fabricate a nanozyme-assisted CDT-PDT combination nanoplatform (TCCHA), which can utilize its cascading catalytic properties to initiate ROS storms, thereby achieving efficient and synergistic anticancer therapy. As shown in Scheme 1, TCCHA nanoparticles were fabricated from 3,3’-dithiobis(propanoic dihydrazide) (TPH), Cu^2+^, and the photosensitizer chlorine e6 (Ce6) via a one-pot self-assembly strategy. By surface modification with aldehyde hyaluronic acid (HAA) and loading of the antivascular drug AL3818, versatile TCCHA nanoparticles were obtained. The versatile TCCHA nanoparticles are expected to exhibit TEM-responsive drug release behavior, triggered by GSH-sensitive disulfide bond-bearing TPH and acid-sensitive coordination. Additionally, these compounds possess effective tumor-targeting capabilities through interactions between hyaluronic acid and the CD44 receptor [32, 33]. In particular, TCCHA nanoparticles could generate ⋅OH and O_2_ by Cu^2+^-mediated Fenton-like and CAT-like activities, respectively. TCCHA could also deplete GSH via disulfide bonding and Cu^2+^-mediated GPx-like activity, thus significantly enhancing the efficacy of CDT and PDT. Moreover, the antivascular drug AL3818 released by TCCHA nanoparticles could hinder tumor angiogenesis, further boosting the therapeutic efficacy of TCCHA nanoparticles. This study provides an innovative strategy for constructing a cascading catalytic nanoplatform and adumbrates an avenue to amplify copper nanoenzyme-based tumor catalytic therapies (Scheme 1).

## Materials and methods

### Materials

Copper(II) chloride dihydrate (CuCl_2_·2H_2_O) was purchased from Sinopharm Chemical Reagent Co., Ltd. (Xi’an, China). Hyaluronic acid (HA, MW: 8000 Da) was obtained from Bloomage Freda Biopharm Co., Ltd. (Shandong, China). Chlorine e6 (Ce6) was purchased from Aladdin Biochemical Technology Co., Ltd. (Shanghai, China). Anlotinib (AL3818) was purchased from Sino Biopharmaceutical Co., Ltd. Methylene blue (MB), terephthalate (TPA) and glutathione (GSH) were purchased from Macklin Biochemical Co., Ltd. (Shanghai, China). A singlet oxygen sensor green (SOSG) fluorescent probe was obtained from Beyotime Biotechnology Co., Ltd. (Shanghai, China). Dulbecco’s modified Eagle’s medium (DMEM), penicillin‒streptomycin, fetal bovine serum (FBS) and 0.25% (w/v) trypsin were obtained from Gibco (USA). A live/dead cell double staining kit (Calcein AM/PI), cell counting kit-8 (CCK-8), annexin V-FITC/PI apoptosis detection kit, Hoechst 33,258 (Hoechst), mitochondrial membrane potential assay kit with JC-1, 2,7-dichlorodihydrofluorescein diacetate (DCFH-DA) kit, LPO fluorescent probe (C11-BODIPY^581/591^) and HIF-1α rabbit polyclonal antibody were purchased from Beyotime Biotechnology Co., Ltd. (Shanghai, China). 3,3’-Dithiobis(propanoic dihydrazide) (TPH) and aldehyde hyaluronic acid (HAA) were synthesized according to our previously reported methods [34, 35]. ^1^H NMR spectra of TPH (DMSO-*d*6, Bruker 400 MHz, δppm): 2.50 (t, 4 H, CO-CH_2_-C**H**_2_), 2.85 (t, 4 H, CO-C**H**_2_-CH_2_), 4.20 (s, 4 H, N**H**_2_-NHCO), and 9.08 (s, 2 H, NH_2_-N**H**CO).

### Preparation of TCC nanoparticles

To prepare the TCC nanoparticles, 2 mL of TPH solution (23.8 mg/mL) was mixed with 1 mL of Ce6 solution (1 mg/mL, dissolved in DMSO) and stirred at 25 °C for 2 h. Subsequently, the mixture was immediately supplemented with 85 µL of CuCl_2_ solution (100 mg/mL) and vigorously stirred for 3 min. Then, the mixed solutions were gently stirred at 25 °C for 24 h. The formed TCC nanoparticles were washed three times with water by centrifugation (18 000 rpm, 10 min), dispersed in water and stored at 4 °C until use.

### Surface modification of TCC nanoparticles

Two milliliters of AHA solution (10 mg/mL) was mixed with 3 mL of TCC nanoparticle suspension under vigorous stirring. After reacting at room temperature for 24 h, the surface-modified TCC (called TCCH) nanoparticles were obtained by centrifugation (18 000 rpm, 10 min), redispersed in water and stored at 4 °C until use.

### Preparation of TCCHA nanoparticles

TCCHA nanoparticles were obtained through a Schiff base reaction. Briefly, 1 mL of 1 mg/mL AL3818 solution was added dropwise to 3 mL of the as-prepared TCCH suspension. After stirring for 24 h, the TCCHA particles were collected via centrifugation at 18,000 rpm for 10 min and washed three times with water. The obtained TCCHA nanoparticles were dispersed in water and stored at 4 °C until use.

### Characterization

UV-vis absorption spectra were recorded by a TU-1810 spectrophotometer (Purkinje General Instrument Co. Ltd. Beijing, China). A JEM-200CX transmission electron microscope (TEM, Nippon Electric Co. Ltd. Japan) was used for morphological analysis of the nanoparticles. The size distribution and zeta potential of the nanoparticles were measured using a Zetasizer Nano ZS90 dynamic light scattering (DLS) instrument (Malvern Instruments, UK). High-performance liquid chromatography (HPLC) data was recorded to determine the amount of released AL3818 (E2695, Waters Corporation, USA). Laser irradiation experiments were conducted using a MW-GX-660 660 nm near-infrared (NIR) laser (Changchun Laser Optoelectronics Technology Co., Ltd., China). In vitro cell fluorescence imaging was performed using a TCS-SP8 confocal laser scanning microscope (CLSM, Leica, Germany) or a fluorescence microscope (BX51, Olympus, Japan). Flow cytometric analysis was performed on a FACS Canto II flow cytometer (BD Biosciences, USA). An IVIS Lumina imaging system (PerkinElmer, America) was used for real-time NIR fluorescence imaging in vivo. To study the stability of the TCCHA nanoparticles, the nanoparticles were dispersed in water, phosphate buffered saline (PBS), saline or cell culture medium (DMEM) for seven days to test their hydrodynamic size.

### In Vitro release study

The drug release behaviors of Ce6 and AL3818 from the TCCHA nanoparticles were evaluated under different pH conditions. Briefly, a TCCHA suspension (5.0 mg/mL, 2.0 mL) was placed in a dialysis bag (MWCO: 1000 Da). The dialysis bag was then immersed in PBS solutions with different pH values (pH 7.4, pH 6.5, pH 5.0). Thereafter, the sample was incubated in a shaker at 37 °C with constant shaking at 100 rpm. At specified time intervals, 2 mL of release medium was removed, and the same volume of fresh medium was immediately added. The release amounts of Ce6 and AL3818 were determined using HPLC.

### Functional characteristics of the TCCHA nanoparticles

#### Fenton-like activity of the TCCHA nanoparticles

To detect the Fenton-like activity of the TCCHA nanoparticles, MB, TMB and TPA were used as indicators. For the MB method, a TCCHA nanoparticle suspension (20 µg/mL), H_2_O_2_ (10 mM) and MB (10 µg/mL) were mixed in deionized water and incubated on a 37 °C shaker for 2 h. Then, the absorption spectrum of the mixture was recorded using a UV‒vis-NIR spectrometer at 20-minute intervals. Similarly, for the TMB assay, a TCCHA nanoparticle suspension (20 µg/mL), H_2_O_2_ (10 mM) and TMB (5 µg/mL) were mixed in deionized water for 10 min. Afterwards, the mixture was removed for UV‒Vis analysis. For the TPA test, TPA (5 µg) was first dissolved in NaOH solution (2 mM, 1 mL) to prepare the TPA working solution. Then, 100 µL of TCCHA nanoparticle suspension (20 µg/mL) and H_2_O_2_ (10 mM) were added to the TPA working solution. After 5 min, the fluorescence intensity of the test solution was recorded by a fluorescence spectrophotometer (λ_ex_ = 310 nm).

#### O_2_ generation

0.1 mL of TCCHA nanoparticle suspension (500 µg/mL) was mixed with 5 mL of H_2_O_2_ solution (10 mM). Subsequently, the O_2_ generated from the mixed solution was monitored by a portable dissolved oxygen meter.

#### Singlet oxygen (^1^O_2_) generation

A singlet oxygen sensor green (SOSG) probe was used to determine the generation of ^1^O_2_ in cells. First, the SOSG working solution was prepared in accordance with the manufacturer’s instructions. Subsequently, the TCCHA nanoparticle suspension was added to the SOSG working solution. The resulting mixture was then exposed to 660 nm laser irradiation for 3 min at a power density of 0.5 W/cm^2^. The production of ^1^O_2_ was assessed by measuring the fluorescence intensity. To induce a hypoxic condition, nitrogen gas was continuously introduced to the working solution for 10 min, followed by the addition of 10 mM H_2_O_2_.

#### GSH consumption

The consumption of GSH was assessed using the DTNB method. Briefly, DTNB solution (2.5 mg/mL, 200 µL), TCCHA suspension (200 µg/mL, 100, 250, 500 or 1000 µL), and GSH solution (5 mM, 50 µL) were added to a centrifuge tube, after which the reaction system was diluted to 4.0 mL with water. Subsequently, the resulting mixture was placed on a shaking Table (37 ℃, 150 rpm) for 30 min, followed by centrifugation to remove any precipitate. Finally, the relative content of GSH was determined by measuring the absorbance of the supernatant at 412 nm using a UV‒vis-NIR spectrometer.

### In vitro cell study

Four cell lines were used, including human hepatoma HepG2 cells, mouse hepatoma Hepa1-6 and H22 cells, and normal mouse fibroblast L929 cells. L929 cells and HepG2 cells were obtained from the Medical Center of Xiʼan Jiaotong University (Xiʼan, China), and H22 and Hepa1-6 cells were purchased from the National Collection of Authenticated Cell Cultures (Shanghai, China). The cells were cultured in DMEM supplemented with 10% FBS and 1% penicillin‒streptomycin. The cells were maintained in a 5% CO_2_ atmosphere at 37 °C and routinely subcultured.

#### In Vitro cellular uptake

The uptake of TCCHA by HepG2 cells was assessed using both confocal laser scanning microscopy (CLSM) and flow cytometry. HepG2 cells were initially seeded in confocal dishes at a density of 1 × 10^5^ cells per dish and incubated in 1 mL of culture medium for 24 h. Subsequently, the cells were incubated with 25 µg/mL TCCHA for 1, 4–8 h. Afterwards, the cells were stained with 5 µg/mL Hoechst 33342 according to the manufacturer’s instructions and then observed via CLSM. In addition, the intracellular Ce6 concentration in HepG2 cells was quantitatively analysed via flow cytometry. Typically, HepG2 cells were seeded into 6-well plates and incubated for 24 h. After being treated as described above, the cells were collected for flow cytometry analysis. To verify whether the HA moiety possessed active targeting ability, a competitive binding experiment was performed. This involved pretreating the cells with 1 mg/mL free HA before coculturing them with the TCCHA nanoparticles. An excess amount of free HA was used to block the CD44 receptor during coculture.

#### In vitro cytotoxicity

First, the cytotoxicity of the TCCHA nanoparticles on HepG2, Hepa1-6, and L929 cells was assessed. The cells were seeded into 96-well plates at a density of 1 × 10^4^ cells/well and cultured for 24 h. Then, the cells were subsequently incubated with various concentrations of TCCHA nanoparticles (10, 20, 50, 100, or 200 µg/mL) for 24 h, after which cell viability was evaluated by a CCK-8 assay. To investigate the effects of HA-mediated active targeting on the cytotoxicity of the TCCHA nanoparticles, HepG2 cells were pretreated with free HA (1 mg/mL) for 1 h. Following pretreatment, the cells were incubated with different concentrations of TCCHA (10, 20, 50, 100, or 200 µg/mL) for 24 h. A CCK-8 assay was subsequently performed to assess cell viability. To assess the cytotoxicity of various drug formulations, HepG2 cells were exposed to free AL3818, free Ce6, TCCH, or TCCHA for 8 h. After rinsing with PBS, the cells in the Ce6-containing groups were irradiated using a 660 nm NIR laser at a power density of 0.5 W/cm^2^ for 3 min. Following a 24-hour incubation, cell viability was determined using the CCK-8 assay.

#### Calcein-AM/PI double-staining assay and cell apoptosis assessment

A calcein-AM/PI double-staining assay was performed to visually assess the cytotoxicity of TCCHA. Briefly, HepG2 cells were seeded in 6-well plates at a density of 5 × 10^5^ cells/well. After 24 h of incubation, the cells were treated with PBS, free AL3818, free Ce6, TCCH, or TCCHA at 37 °C for 8 h. Subsequently, the cells in the Ce6-containing groups were irradiated with a 660 nm NIR laser (0.5 W/cm^2^, 3 min). After 4 h, the cells were stained with calcein-AM and PI according to the manufacturer’s protocol and observed using a fluorescence microscope.

Flow cytometry was used to analyse the mechanism of cell death. HepG2 cells were initially seeded in 6-well plates at a density of 5 × 10^5^ cells/well for 24 h. Subsequently, the cells were treated according to the procedures described for the calcein-AM/PI double-staining assay. After that, the cells were treated with an annexin V-FITC/PI apoptosis detection kit, and flow cytometry analysis was conducted.

#### Intracellular GSH consumption

HepG2 cells were seeded into a 6-well plate at a density of 5 × 10^5^ cells/well for 24 h. Then, the cells were treated with either 25 µg/mL or 100 µg/mL TCCHA. After 12 h, the cells were collected, and the intracellular content of GSH was measured using a GSH/GSSG detection kit.

#### Evaluation of intracellular ROS, LPO and H_2_O_2_ levels

HepG2 cells were seeded into confocal dishes at a density of 2 × 10^5^ cells/dish for 24 h. Subsequently, the cells were incubated with PBS, free Ce6, or TCCHA for 8 h. For the ROS assay, the cells were stained with 10 µM DCFH-DA for 30 min according to the manufacturer’s protocol. The cells in the NIR groups were irradiated with a 660 nm laser for 3 min at a power density of 0.5 W/cm^2^. CLSM was used to observe the cells. In addition, the cells were cultured under hypoxic condition for 12 h and then subjected to the same procedures as those described above to measure ROS levels. For the LPO assay, the cells were stained with 5 µM LPO fluorescent probe C11-BODIPY^581/591^ for 30 min and then detected via CLSM.

To examine the level of H_2_O_2_, the cells were treated with different concentrations of TCCHA (25–100 µg/mL) for 12 h. Afterwards, H_2_O_2_ levels were determined by using a fluorometric hydrogen peroxide assay kit.

#### Evaluation of hypoxia relief in Vitro

Immunofluorescence staining was performed to detect intracellular hypoxia relief. HepG2 cells were seeded into confocal dishes at a density of 2 × 10^5^ cells/dish and cultured under hypoxic conditions for 12 h. Subsequently, the cells were treated with either 25–100 µg/mL TCCHA for 2 h. Afterwards, the cells were washed, fixed, and stained with anti-HIF-1α according to the manufacturer’s instructions. CLSM was used to visually observe and determine the level of cellular hypoxia relief. In addition, a hypoxia probe was used to evaluate the hypoxia relief ability of TCCHA according to the manufacturer’s instructions. CLSM was also employed to visually investigate the degree of cellular hypoxia.

### In vivo animal study

Four- to five-week-old BALB/c mice were obtained from the Experimental Animal Center of Xi’an Jiaotong University. All animal experiments were conducted in accordance with protocols approved by the Animal Care and Use Committee of Xi’an Jiaotong University. All necessary ethical regulations were strictly adhered to during the experiments. To evaluate the antitumor effects of TCCHA nanoparticles on human hepatocellular carcinoma, a HepG2 tumor-bearing model was established. One hundred microliters of HepG2 cells (human hepatocellular carcinoma cell line) was subcutaneously injected (1 × 10^7^ cells) into the right flank of each immunodeficient nude mouse. Tumor-bearing mice were treated when the tumor volume reached approximately 100 mm^3^. To further confirm the antitumor effects of TCCHA nanoparticles, an H22 tumor-bearing model was established. H22 tumor cells (a mouse hepatocellular carcinoma cell line) were injected subcutaneously (1 × 10^6^ cells) into the right flanks of each immune-competent BALB/c mouse. The mice were treated when the tumor volume reached approximately 80 mm^3^. The in vivo antitumor experiment was conducted following the same steps as those for HepG2 tumor-bearing BALB/c nude mice. Furthermore, a lung metastatic Hepa1-6 tumor model was established. A tumor lung metastasis model was established through subcutaneous injection of 5 × 10^6^ Hepa1-6 cells into the right flank and intravenous injection of 5 × 10^5^ Hepa1-6 cells separately at day 7 and day 2 before the treatments for development of the primary and lung metastasis tumors, respectively. The mice were treated when the tumor volume reached approximately 100 mm^3^.

#### In vivo fluorescence imaging and biodistribution

To trace the in vivo distribution of TCCHA, 100 µL of 5 mg/mL TCCHA suspension was intravenously injected into tumor-bearing mice. At predetermined time points, the mice were imaged on an IVIS Lumina imaging system. At the indicated time points, the mice were sacrificed, and major organs and tumors were collected for ex vivo fluorescence imaging.

The biodistributions of Cu^2+^ in major organs and tumors were assessed by ICP-MS, and the biodistributions of Ce6 and AL3818 were assessed by HPLC. For ICP-MS analysis, each tumor-bearing mouse was intravenously injected with 100 µL of 5 mg/mL TCCHA and then sacrificed at predetermined time intervals (*n* = 5 per time point). The tumors and main organs were collected, wet-weighed, lyophilized, and ground into powder. Subsequently, the powder samples were predigested with aqua regia overnight, completely digested in open vessels at 140 °C for 2 h, and then cooled to room temperature. Each solution was diluted with water to 10 mL for ICP-MS analysis. For HPLC analysis, the tumors and main organs were collected, wet-weighed, homogenized, extracted with chloroform, and analysed via HPLC.

#### In vivo antitumor efficacy

Tumor-bearing mice were randomly divided into six groups (*n* = 6): (1) PBS (control group), (2) Ce6 + Laser, (3) AL3818, (4) TCCH + Laser, (5) TCCHA, and (6) TCCHA + Laser. The mice were intravenously injected with 100 µL of the above formulations at an equivalent dose of TCCHA (5 mg/kg). At 8 h postinjection, the tumors in the laser treatment groups were irradiated with a 660 nm laser for 5 min at 1.0 W/cm^2^. The irradiation procedure was repeated every 2 days for a total of three times. The tumor volume and body weight were recorded every 2 days. Tumor volume was calculated by the following equation: Volume = (tumor length) × (tumor width)^2^/2. After 14 days, the mice were sacrificed, and the tumors were harvested, weighed and analysed by H&E, TUNEL, Ki67, and CD31 staining. to determine the antitumor efficacy of the TCCHA nanoplatform.

### Statistical analysis

All data were represented as the means ± standard deviations (S.D.). Statistical comparisons were made by unpaired Student’s t test (between two groups) or one-way ANOVA (for multiple comparisons). *ns* indicates no significance. *p < 0.05 was considered statistically significant, whereas **p < 0.01 and ***p < 0.001 were considered highly and extremely significant, respectively. All the data were analysed with SPSS 17.0 software.

## Results and discussion

### Preparation and characterization of TCCHA nanoparticles

**Fig. 1:**
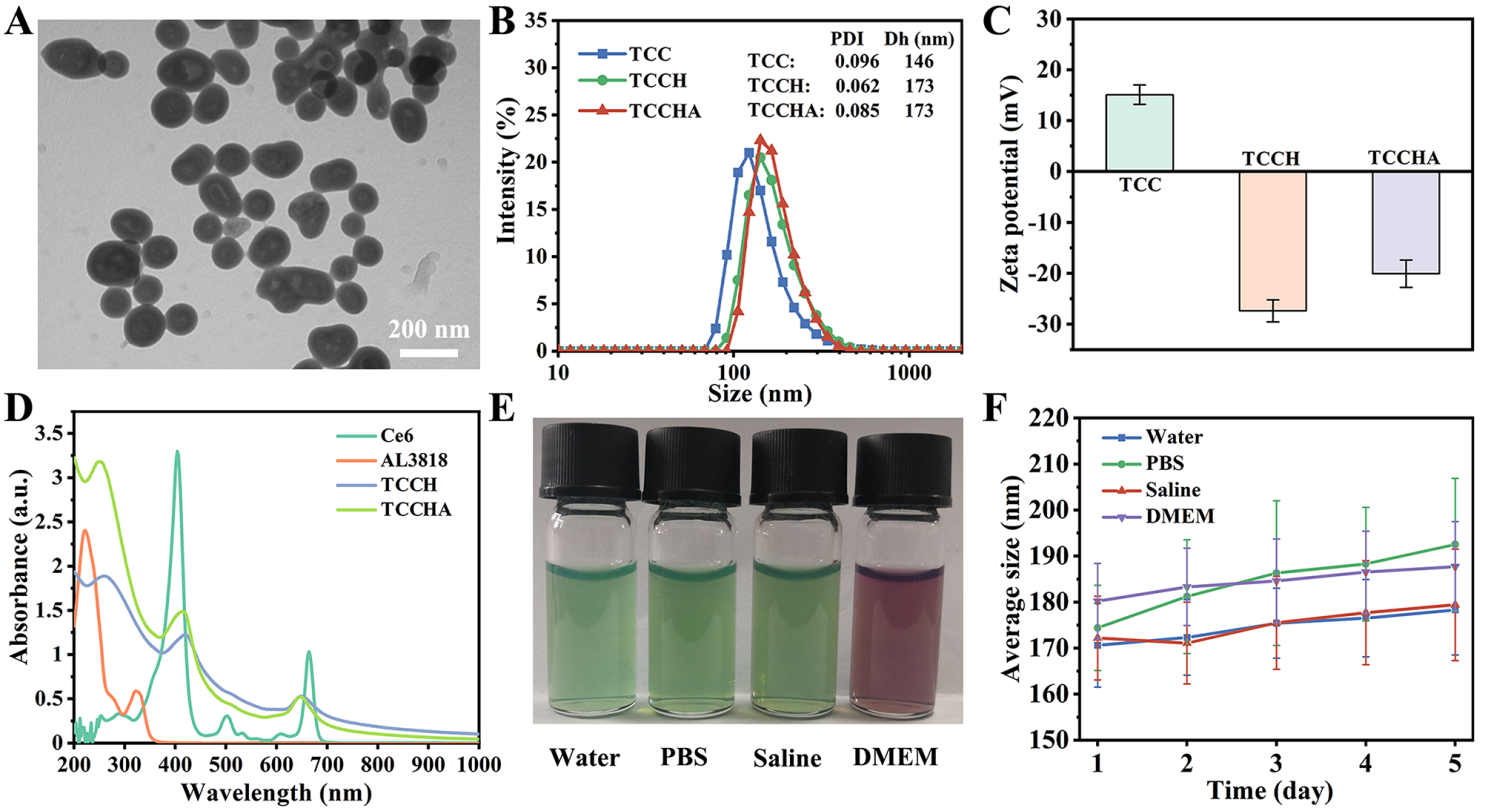
Preparation and characterization of TCCHA nanoparticles. (**A**) TEM images of TCCHA nanoparticles. (**B**) Hydrodynamic size distributions and (**C**) zeta-potentials of TCC, TCCH and TCCHA. (**D**) UV–vis spectra of Ce6 and AL3818 solutions, and TCCH and TCCHA suspensions. (**E**) The dispersion stabilities (from left to right in photograph) and (**F**) size stabilities of TCCHA nanoparticles in water, PBS, saline, and DMEM within 5 days. *n* = 3

A facile self-assembly strategy for the synthesis of TCCHA is illustrated in Scheme 1. TCC nanoparticles were first prepared through simple one-pot self-assembly of TPH, Cu^2+^ and Ce6. Then, the TCCH nanoparticles were modified with HAA to obtain TCCH nanoparticles via electrostatic adsorption and Schiff base reaction between the aldehyde group of HAA and the hydrazide group of TPH. Once again, a Schiff base reaction was used to load AL3818 onto the TCCH, leading to the formation of TCCHA nanoparticles. TEM observations revealed that the obtained TCCHA nanoparticles exhibited a monodisperse near-spherical morphology with an average size of 106 nm (Fig. 1A). The dynamic light scattering (DLS) data showed that the TCC, TCCH, and TCCHA nanoparticles exhibited a single hydrodynamic size distribution peak (Fig. 1B). Specifically, the TCC, TCCH, and TCCHA nanoparticles had hydrodynamic sizes of 157 nm, 176 nm, and 188 nm, respectively, and surface potentials of + 15.1 mV, -27.5 mV, and − 20.1 mV, respectively (Fig. 1B, C). Notably, both the TCCH and TCCHA nanoparticles demonstrated considerably negative zeta potentials, which can be attributed to the modification of HA. This characteristic is beneficial for maintaining stability and prolonging the circulation time of TCCHA [36]. The successful loading of the photosensitizer Ce6 and the antivascular drug AL3818 was confirmed by UV-vis absorption measurements. As shown in Fig. 1D, both TCCH and TCCHA nanoparticles displayed two peaks at 663 nm and 400 nm, which were attributed to the absorption of Ce6. In addition, TCCHA nanoparticles displayed enhanced absorption near 320 and 230 nm compared to TCCH, which should be due to the loading of AL3818. Furthermore, the loading amounts of Ce6 and AL3818 were quantified by UV-vis spectroscopy to be approximately 10.2 wt% and 4.0 wt%, respectively. The dispersion stability of the TCCHA nanoparticles was evaluated in water, PBS, saline, and DMEM. The results (Fig. 1E, F) revealed that the TCCHA nanoparticles exhibited negligible changes in size and maintained a stable dispersion in the aforementioned media within 5 days, indicating their excellent biostability.

### Cascading catalytic properties of TCCHA nanoparticles for realizing ROS storms

**Fig. 2:**
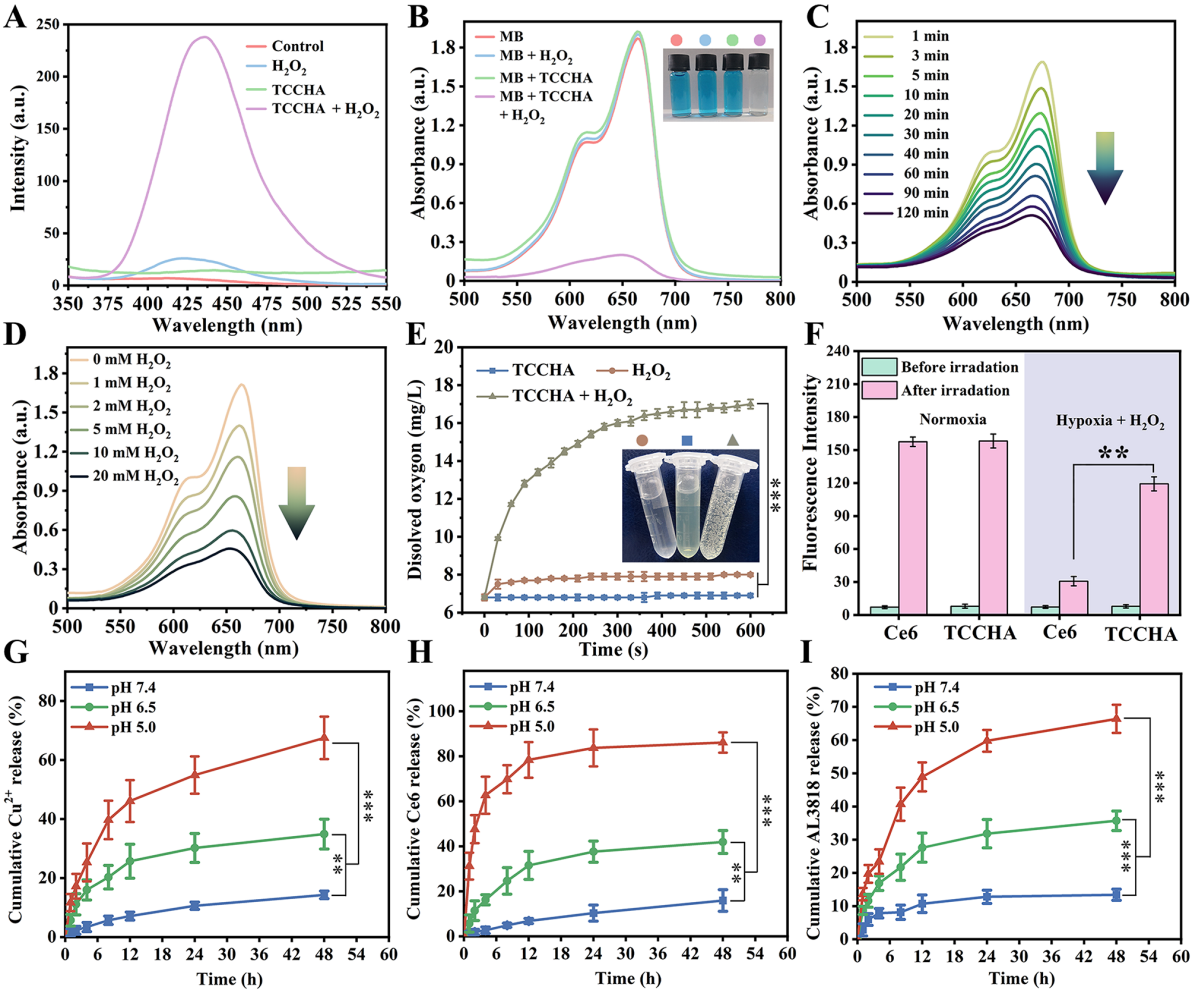
Physicochemical properties of the TCCHA nanoparticles. (**A**) Fluorescence spectra of TPA solutions and (**B**) UV-vis spectra of MB solutions after incubation with pristine H_2_O_2_, TCCHA and H_2_O_2_/TCCHA mixed solutions. UV-vis spectra of MB solutions incubated with H_2_O_2_/TCCHA mixed solutions at (**C**) different time intervals and (**D**) different H_2_O_2_ concentrations. (**E**) Changes in the dissolved oxygen concentration in pristine H_2_O_2_, TCCHA and H_2_O_2_/TCCHA mixed solutions. *n* = 3, *** *p* < 0.001. (**F**) Fluorescence intensities of the SOSG probe after incubation with Ce6 or TCCHA in the presence of H_2_O_2_ under normoxic/hypoxic conditions before and after NIR laser irradiation. *n* = 3, ** *p* < 0.01. Cumulative release profiles of (**G**) Cu^2+^, (**H**) Ce6 and (**I**) AL3818 from TCCHA nanoparticles dispersed in different pH buffers (pH 7.4, pH 6.5, pH 5.0). *n* = 3, ** *p* < 0.01, *** *p* < 0.001

Recently, ROS, including singlet oxygen (^1^O_2_) or hydroxyl radicals (⋅OH), have been shown to cause strong oxidative lethality that can effectively kill tumor cells. To evaluate the generation of ⋅OH, the Fenton-like catalytic activity of the TCCHA nanoparticles was studied. Terephthalic acid (TPA), methylene blue (MB), and 3,3′,5,5′-tetramethylbenzidine (TMB) were used as indicators. It is well established that ⋅OH can effectively oxidize these indicators with different optical properties. Nonfluorescent TPA can be oxidized by ⋅OH to form fluorescent 2-hydroxy terephthalic acid (TPA-OH). The fluorescence intensity (Fig. 2A) observed in the presence of H_2_O_2_ or TCCHA alone was almost negligible. However, this fluorescence signal significantly increased when TCCHA was combined with H_2_O_2_, indicating the successful generation of TPA-OH. This can be attributed to the catalytic conversion of H_2_O_2_ into ⋅OH by TCCHA. Subsequently, MB was employed for further confirmation of ⋅OH generation. In the presence of TCCHA and H_2_O_2_, the absorption of MB decreased significantly (Fig. 2B). Furthermore, as time progressed, the absorbance of the MB solution in the presence of TCCHA and H_2_O_2_ decreased significantly (Fig. 2C). After 2 h, the MB was nearly completely degraded, confirming the ability of the TCCHA nanoparticles to catalyze H_2_O_2_ to produce ⋅OH. Additionally, higher H_2_O_2_ concentrations accelerated the degradation of MB (Fig. 2D), further indicating a positive correlation between the amount of ⋅OH produced and the H_2_O_2_ concentration. Similarly, colorless TMB is easily oxidized by ⋅OH to blue-oxidized TMB (oxTMB). In the presence of TCCHA and H_2_O_2_, the color of the TMB solution changed from colorless to navy blue, and a strong characteristic absorption peak of oxTMB at 650 nm was observed at the same time, confirming the production of ⋅OH (Figure S1). Therefore, TCCHA nanoparticles hold great promise for CDT.

The photosensitizer Ce6 has been extensively used in near-infrared laser-activated PDT [37, 38]. However, the efficacy of Ce6-mediated PDT is often restricted by the hypoxic TME. Therefore, we examined the catalase-like (CAT) catalytic activity of TCCHA nanoparticles to assess the production of O_2_. As shown in Fig. 2E, the dissolved oxygen levels in the TCCHA + H_2_O_2_ solution group were significantly higher than those in the other groups. Specifically, after approximately 8 min, the O_2_ concentration in the TCCHA + H_2_O_2_ solution reached 17.1 mg/L, whereas the O_2_ concentration in the pristine H_2_O_2_ solution and TCCHA suspension were 8.0 mg/L and 6.9 mg/L, respectively. These results suggested that TCCHA displays superior CAT catalytic activity. Notably, excessive O_2_ bubbles were observed in the TCCHA + H_2_O_2_ solution, accompanied by a color change from colorless to yellow due to the formation of an intermediate product of CuO_2_ [39]. In addition, singlet oxygen (^1^O_2_) generation from TCCHA was examined using a singlet oxygen sensor green (SOSG) probe as an indicator under laser irradiation (660 nm). As shown in Fig. 2F, the ^1^O_2_ generation efficiency of the TCCHA nanoparticles was comparable to that of free Ce6 under normoxia condition, suggesting that the formation of TCCHA nanoparticles did not affect the photodynamic efficiency of Ce6. Interestingly, under hypoxia condition, the fluorescence intensity of the TCCHA nanoparticles was significantly higher than that of free Ce6 (approximately 3.9 times higher). This enhancement can be attributed to the Fenton-like reaction mediated by the TCCHA nanoparticles, which provided a greater availability of O_2_ molecules for PDT to produce ^1^O_2_.

GSH, a crucial antioxidant in cancer cells, plays a significant role in eliminating excessive ROS, including ⋅OH and ^1^O_2,_ to maintain dynamic intracellular redox balance [40]. Excitingly, TCCHA was expected to have glutathione peroxidase-like (GPx) catalytic activity that can remove GSH due to the presence of disulfide bond and Cu^2+^ in the particles. The DTNB method was used to quantify the depletion of GSH. As shown in Figure S2, the absorption at 412 nm decreased with increasing TCCHA concentration, verifying that TCCHA could consume GSH. Additionally, the suspension of GSH-treated TCCHA emitted strong pink fluorescence under UV irradiation (Figure S3), which was due to the reduction of Cu^2+^ to Cu^+^ by GSH. The above results confirmed the GPx and CAT catalytic activities of TCCHA. CAT activity enables TCCHA to self-supply O_2_ to alleviate the hypoxic TME and enhance the efficacy of PDT, and GPx activity endows TCCHA with GSH depletion capability to disrupt the intracellular oxidative defense system. Collectively, the cascading catalytic activities of TCCHA enable it to generate ROS storms to enhance the efficacy of CDT-PDT.

It is worth mentioning that the feasibility of the clinical application of TCCHA nanoparticles is determined by whether drugs can be rapidly released at the tumor site. Therefore, we investigated the release profiles of TCCHA nanoparticles in PBS buffer at pH 5.0, 6.5, and 7.4. As depicted in Fig. 2G-I, the TCCHA nanoparticles demonstrated exceptional stability at pH 7.4, with only 14.3%, 16.7% and 12.8% of the Cu^2+^, Ce6 and AL3818 being released within 48 h, respectively. In contrast, TCCHA exhibited a distinct burst drug release pattern, with cumulative release percentages of 67.5% for Cu^2+^, 86.2% for Ce6 and 65.2% for AL3818 at pH 5.0. Additionally, a moderate drug release pattern was observed at pH 6.5. The observed acid-sensitive drug release pattern can be attributed to the fact that the TCCHA nanoparticles are assembled from acid-sensitive coordination bonds. Additionally, the presence of a disulfide bond in TCCHA rendered it GSH-responsive ability. Therefore, we examined the release profiles of TCCHA nanoparticles in PBS and at various concentrations of GSH (1, 5, and 10 mM). The cumulative release percentages increased with increasing GSH concentration (Figure S4-6). For instance, in the case of 10 mM GSH, the release percentages of Cu^2+^, Ce6 and AL3818 increased to 72.6%, 83.3% and 76.9%, respectively, at 48 h, which were much greater than those observed under the other conditions.

### In vitro cellular uptake and therapeutic efficacy

**Fig. 3:**
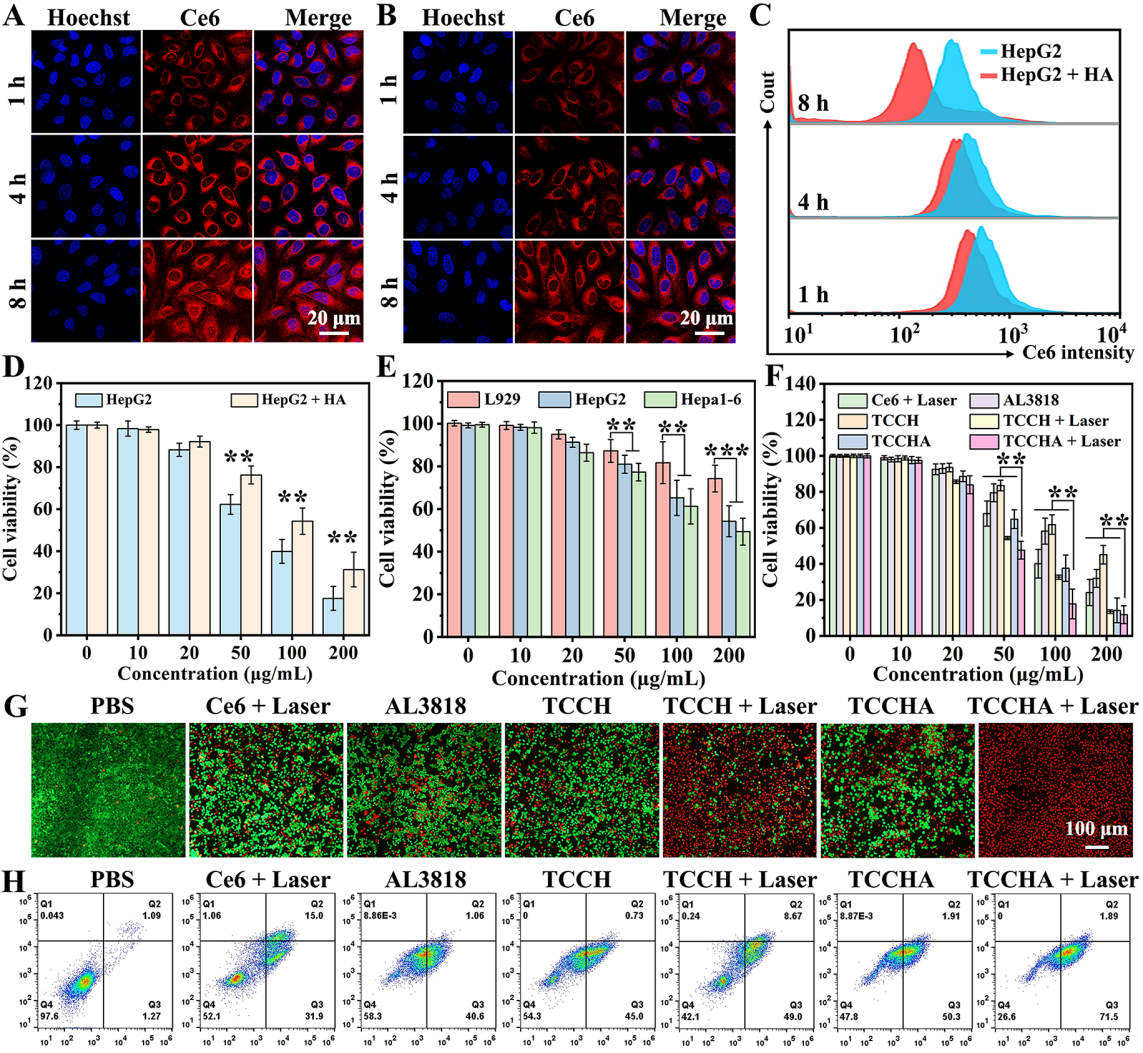
Cellular uptake and cytotoxicity. (**A**) CLSM images of HepG2 cells treated with TCCHA for 1, 4 and 8 h (Blue: Hoechst stained nuclei. Red: Ce6). (**B**) CLSM images of HepG2 cells treated with TCCHA for 1, 4 and 8 h after treatment with 1 mg/mL free HA for 24 h (Blue: Hoechst stained nuclei. Red: Ce6). (**C**) Flow cytometric results of the relative changes of intracellular Ce6 levels in HepG2 cells group or HA pretreatment HepG2 cells group (HepG2 + HA) for 1, 4, and 8 h. (**D**) Viabilities of in HepG2 cells group or HA pretreatment HepG2 cells group (HepG2 + HA) with different concentrations of TCCHA for 24 h. *n* = 3, ** *p* < 0.01. (**E**) L929, HepG2 and Hepa1-6 cells after treatment with different concentrations of TCCHA for 24 h. *n* = 3, ** *p* < 0.01, *** *p* < 0.001. (**F**) Viabilities of HepG2 cells after treatment with different formulations of Ce6 + Laser, AL3818, TCCH, TCCHA, TCCH + Laser, or TCCHA + Laser for 24 h (660 nm Laser: 0.5 W/cm2, 3 min). *n* = 3, ** *p* < 0.01. (**G**) Fluorescence images of calcein-AM/PI double-stained HepG2 cells after treatment with different formulations (Red: PI, dead cells. Green: calcein-AM, living cells). (**H**) Flow cytometry of apoptosis in HepG2 cells after treatment with different formulations

The efficient uptake of TCCHA nanoparticles by tumor cells is essential for its antitumor effect. To verify the targeting capability of TCCHA, a competitive binding experiment was used to examine the cellular internalization of the TCCHA nanoparticles in HepG2 cells. As shown in Fig. 3A and B, CLSM images demonstrated that the fluorescence intensity of intracellular Ce6 increased over time, indicating enhanced uptake of the TCCHA nanoparticles by HepG2 cells. Notably, the red Ce6 fluorescence intensity observed in the free HA pretreatment group (Fig. 3B) was significantly weaker than that in the group without HA pretreatment (Fig. 3A), mainly because HA pretreatment blocks the CD44 receptor [33]. These findings further suggest that TCCHA nanoparticles exhibit greater tumor-targeting ability mediated by the HA-CD44 receptor interaction. Additionally, flow cytometry was used to quantitatively investigate the cellular uptake of the TCCHA nanoparticles in HepG2 cells. As shown in Fig. 3C, the flow cytometric results were consistent with the CLSM observations and indicated the continuous uptake of the TCCHA nanoparticles by the cells. The corresponding statistical analysis is displayed in Figure S7. Notably, after 8 h of incubation, the fluorescence intensity in the HepG2 group (without HA pretreatment) was approximately 1.25-fold greater than that in the HepG2 + HA group (with HA pretreatment), further confirming the superior targeting ability of the TCCHA nanoparticles.

**Fig. 4:**
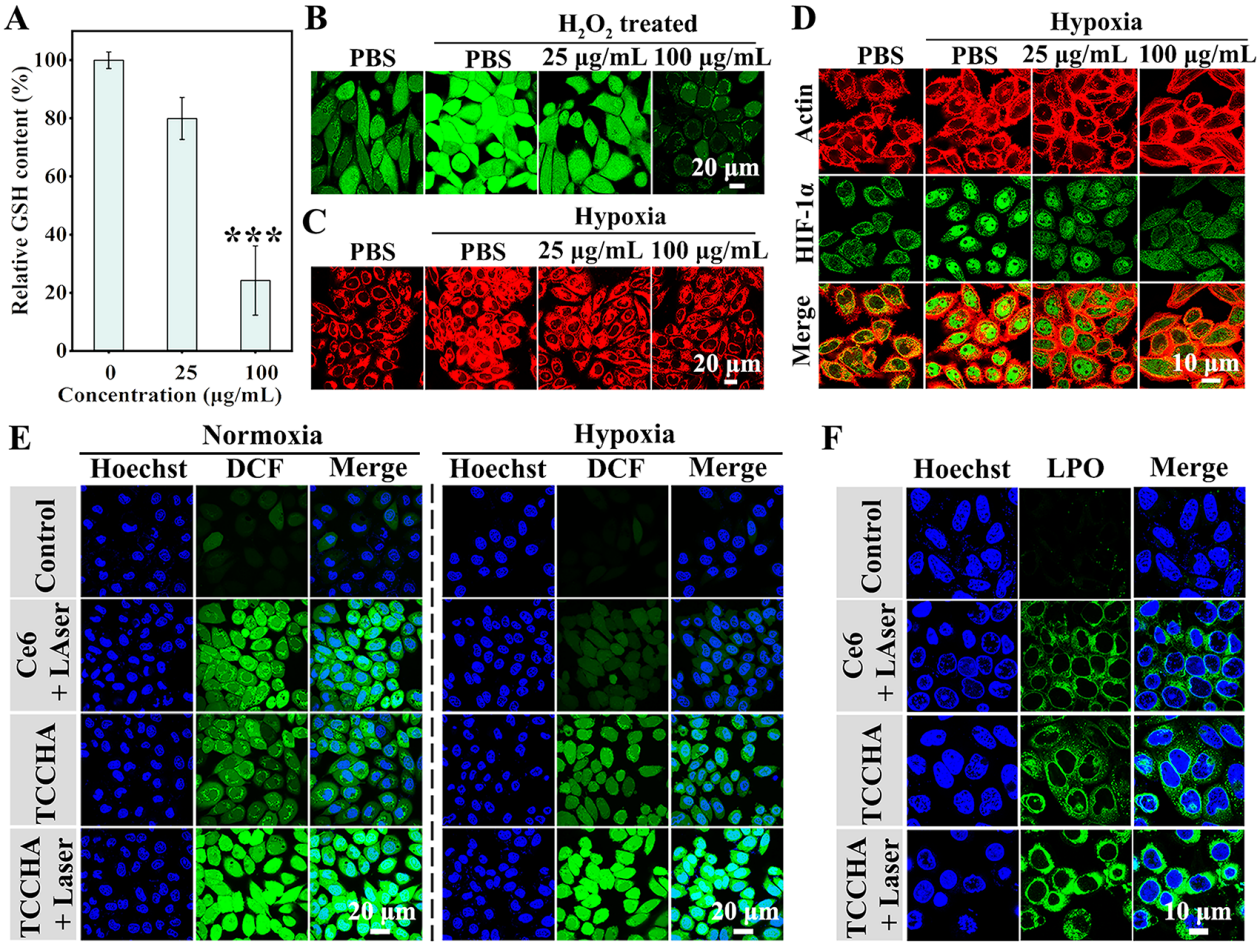
Studies on the antitumor mechanism of TCCHA nanoparticles. (**A**) The relative GSH contents in HepG2 cells after various treatments. *n* = 3, ***p < 0.001. (**B**) H_2_O_2_ levels in HepG2 cells after various concentrations of TCCHA treatment with or without H_2_O_2_ pretreatment (Green: H_2_O_2_ probe). (**C**) Fluorescence images of HepG2 cells after various concentrations of TCCHA treatment with or without hypoxia pretreatment (Red: Hypoxia probe). (**D**) Levels of HIF-1α after various concentrations of TCCHA treatment with or without hypoxia pretreatment. (**E**) CLSM images reflecting ROS generation in HepG2 cells treated with various formulations of Ce6 + Laser, TCCHA, or TCCHA + Laser for 8 h with or without hypoxia pretreatment. (**F**) CLSM images reflecting LPO generation in HepG2 cells after different treatments

**Fig. 5:**
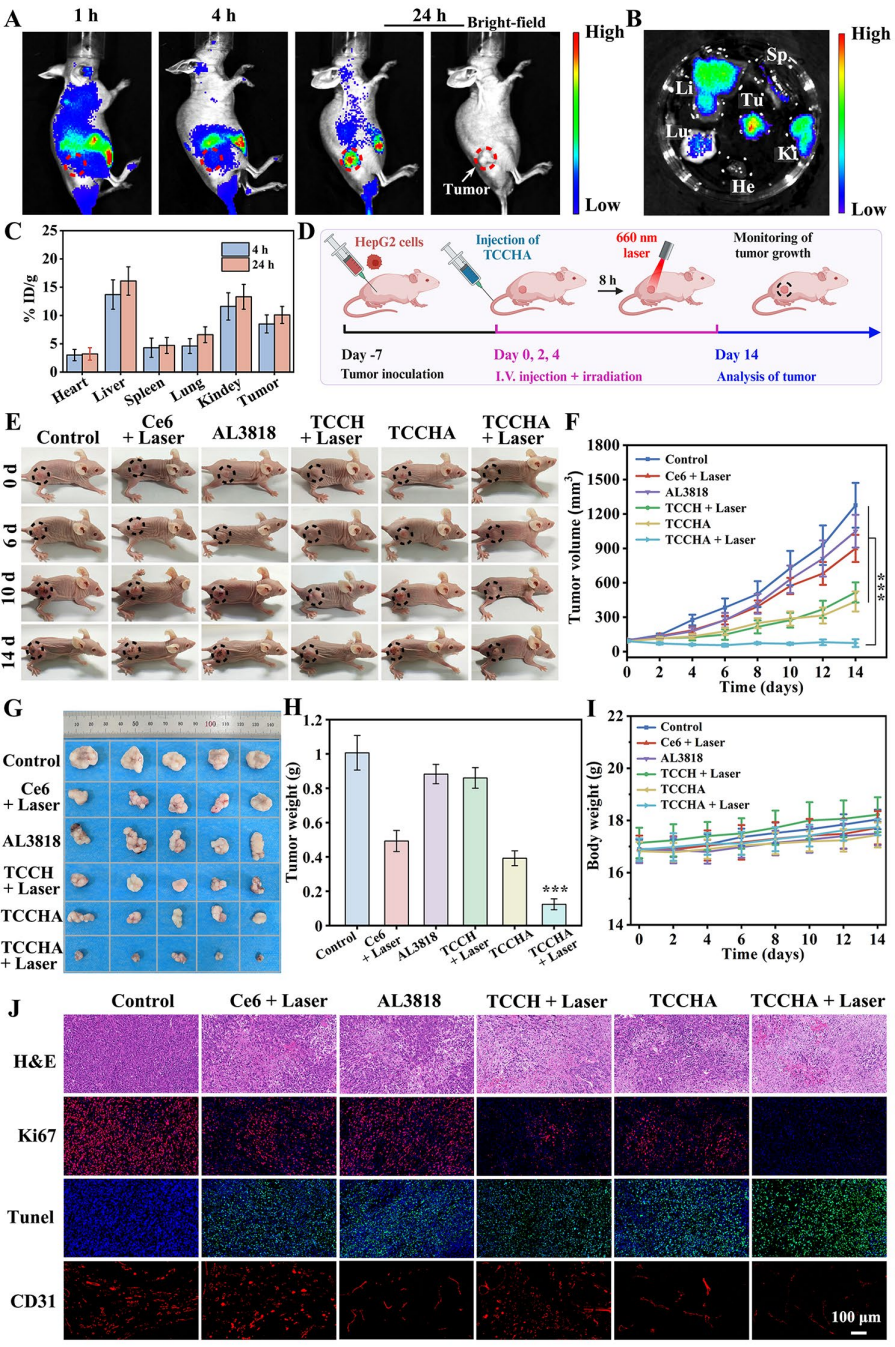
Biodistribution and antitumor efficacy. (**A**) In vivo fluorescence images of HepG2 tumor-bearing mice at 1 h, 4 h, and 24 h after intravenous injection of TCCHA. Tumor sites were marked with red circles. (**B**) Ex vivo fluorescence images of tumors (Tu) and major organs, including heart (He), liver (Li), spleen (Sp), lung (Lu) and kidney (Ki), collected at 24 h postinjection. (**C**) Quantitative biodistribution of TCCHA in major organs and tumors at different time points postinjection, as determined by ICP-MS analysis of Cu content. (**D**) Therapeutic schedule of TCCHA nanoparticle-mediated anticancer treatment using a HepG2 tumor-bearing mouse model. (**E**) Digital photographs of mice taken at 0 d, 6 d, 10 d and 14 d after treatment with different formulations of Ce6 + laser, AL3818, TCCH + laser, TCCHA, or TCCHA + laser. The tumor sites were marked with black circles. (**F**) tumor growth curves of mice in different groups during treatment. *n =* 6, ***p < 0.001. (**G**) Digital photographs of the corresponding tumor images after 14 days of treatment. (**H**) tumor weight of HepG2 tumor-bearing mice after various treatments. *n* = 6, ***p < 0.001. (**I**) Body weights of mice that received different treatments within 14 days. *n =* 6. (**J**) H&E, immunofluorescence staining of Ki67, TUNEL and CD31 images of tumor sections from different treatment groups after 14 days

Thereafter, the anticancer efficacy of TCCHA was investigated in vitro. We first assessed the cytotoxicity of TCCHA to HepG2 cells without HA pretreatment (HepG2 group) and to HepG2 cells with HA pretreatment (HepG2 + HA group) using the CCK8 assay. As shown in Fig. 3D, the cell viability in the two groups decreased progressively in a concentration-dependent manner. Notably, the HepG2 group exhibited higher cytotoxicity compared to the HepG2 + HA group at the same concentrations. This observation agreed with the cellular uptake results, indicating that increased internalization of TCCHA nanoparticles by tumor cells could lead to a more potent killing efficacy. Moreover, we examined the cytotoxicity of the TCCHA nanoparticles to L929 normal cells, HepG2 and Hepa1-6 tumor cells. As illustrated in Fig. 3E, the viability of L929 cells treated with the same concentrations of TCCHA was significantly higher compared to that of HepG2 and Hepa1-6 cells, further implying that TCCHA nanoparticles could more efficiently kill tumor cells. We further assessed the cell-killing capability of various formulations against HepG2 cells. (Fig. 3F). The cells treated with TCCHA nanoparticles exhibited higher cytotoxicity than did those treated with single therapies, including Ce6 + laser (PDT), AL3818 (antiangiogenic therapy) or TCCH (CDT), which could be attributed to the combined effect of CDT and antiangiogenic therapy. Moreover, TCCHA + laser exhibited the most robust cell-killing ability due to the synergistic effect of the combined antiangiogenic therapy CDT-PDT. To further assess the cell-killing effect, we conducted a live/dead cell staining assay. As shown in Fig. 3G, compared with those in the other treatment groups, the cells in the TCCHA + Laser group exhibited a higher percentage of cell death. This was evident from the faint green fluorescence and strong overall red fluorescence, which were consistent with the findings in Fig. 3F. These results suggest that TCCHA + laser treatment had a superior cell-killing effect. Furthermore, an annexin V/PI apoptosis assay was conducted to evaluate the apoptosis-inducing ability of the TCCHA nanoparticles. Similarly, the TCCHA + Laser group exhibited the highest percentage of apoptotic cells (73.39%) (Fig. 3H). The quantitative data (Figure S8) clearly indicated that the apoptosis rate in the TCCHA + Laser group was significantly greater than that in the control group (21.4-fold), Ce6 + Laser group (1.5-fold), AL3818 group (1.7-fold), TCCH group (1.6-fold), TCCH + Laser group (1.3-fold), and TCCHA group (1.4-fold). These results can also be attributed to the synergistic antiangiogenic therapy-CDT-PDT. These findings highlight that TCCHA nanoparticles have an excellent ability to induce tumor cell apoptosis.

### Mechanism of TCCHA nanoparticle-induced cell death

Inspired by the excellent in vitro antitumor efficacy of TCCHA, we further explored its antitumor mechanism. A cascading catalytic ROS therapy mechanism was proposed and validated through multiple complementary methods. GSH is the most crucial ROS scavenger in cancer cells and plays a crucial role in maintaining intracellular redox homeostasis [40]. Since the antitumor effect of TCCHA nanoparticles relies on ROS therapy, including ⋅OH-based CDT and ^1^O_2_-based PDT, intracellular GSH levels strongly affect the cytotoxicity of TCCHA nanoparticles. Therefore, we first studied intracellular GSH depletion (Fig. 4A). The results demonstrated a significant reduction in the intracellular GSH content with increasing TCCHA concentration, which indicated that the TCCHA nanoparticles could effectively eliminate intracellular GSH. This is attributed to both the continuous redox reaction between GSH and Cu^2+^ and the thiol-disulfide bond exchange reaction between GSH and TPH. To demonstrate the H_2_O_2_ degradation capability of TCCHA, the intracellular H_2_O_2_ content in HepG2 cells was measured by using an H_2_O_2_ probe. CLSM images (Fig. 4B) showed that H_2_O_2_ pretreatment significantly elevated intracellular H_2_O_2_ levels compared to those in the nonpretreated PBS group, as evidenced by the brighter green fluorescence observed using the H_2_O_2_ probe. Interestingly, once the cells were treated with TCCHA, the intracellular green fluorescence weakened, and a higher concentration of TCCHA resulted in weaker fluorescence signals, verifying the robust ability of TCCHA to consume H_2_O_2_ for Fenton-like reactions in cancer cells. The quantitative data (Figure S9) clearly indicated that the intracellular level of H_2_O_2_ in the PBS group was approximately 17.5-fold greater than that in the 100 µg/mL TCCHA treatment group. These findings confirmed that the TCCHA nanoparticles could effectively decompose H_2_O_2_.

The CAT catalytic ability of TCCHA enables the generation of O_2_ through a catalytic reaction. The produced O_2_ has the potential to alleviate tumor hypoxia. A hypoxia detection kit was utilized to visually assess the efficacy of TCCHA in alleviating tumor hypoxia. Compared with those cultured under normoxic conditions, HepG2 cells cultured under hypoxic conditions emitted more intense red fluorescence (Fig. 4C), demonstrating the successful establishment of a hypoxic intracellular microenvironment. Excitingly, the hypoxic cells treated with TCCHA exhibited significantly reduced fluorescence with increasing TCCHA concentration, indicating successful alleviation of hypoxia. This was attributed to the CAT catalytic ability of the TCCHA nanoparticles to catalyze H_2_O_2_ to produce O_2_. In addition, hypoxia-inducible factor-1α (HIF-1α) is a transcription factor that serves as a key biomarker for hypoxia and is stable only under hypoxic conditions. In the cytoplasm, HIF-1α binds to HIF-1β to form a transcriptionally active dimer, which is transported to the nucleus once the cells become hypoxic [41, 42]. To further examine the ability of TCCHA nanoparticles to relieve hypoxia, we used fluorescence staining to measure HIF-1α expression. As shown in Fig. 4D, slight green fluorescence was observed in the cytoplasm of cells cultured under normoxic conditions, whereas bright fluorescence appeared in the nuclei under hypoxia. After the cells were treated with TCCHA, the intensity of the green fluorescence signal decreased significantly, indicating the downregulation of HIF-1α and hypoxia relief. Furthermore, we observed a dose-dependent effect of TCCHA on the degree of hypoxia relief, with higher concentrations resulting in greater downregulation of HIF-1α. The quantitative data (Figure S10) revealed that the fluorescence intensity of the hypoxic cells in the PBS group were 1.66- and 3.76-fold higher than those in the 25 µg/mL and 100 µg/mL TCCHA-treated groups, respectively. Considering that the photosensitizer Ce6 requires O_2_ to generate ^1^O_2_ for PDT, these results suggested that TCCHA nanoparticles not only have great potential for alleviating hypoxia in the TME but also for enhancing the efficacy of ^1^O_2_-based PDT.

Thereafter, we measured intracellular ROS levels (including ⋅OH and ^1^O_2_) using the DCFH-DA probe under normoxic and hypoxic conditions. The DCFH-DA probe can be oxidized by ROS to form fluorescent DCF, which indicates the level of ROS. CLSM images (Fig. 4E) showed that HepG2 cells in the TCCHA + Laser group exhibited significantly stronger DCF fluorescence signal compared to the other groups, regardless of whether the conditions were normoxic or hypoxic. Furthermore, compared with that in the hypoxic group, the DCF fluorescence in the treatment group under normoxic conditions was much stronger. These results indicate that the production of ROS is dependent on the level of O_2_. This is mainly because the production of ^1^O_2_ requires the presence of O_2_. Notably, quantitative analysis (Figure S11) revealed that under normoxic condition, the ROS fluorescence intensity in the TCCHA + Laser group was approximately 2.6-fold greater than that in the TCCHA group and 1.8-fold greater than that in the Ce6 + Laser group. These observations confirmed that TCCHA nanoparticles combined with laser irradiation have a greater ability to generate intracellular ROS. The LPO probe was subsequently utilized to detect the degree of lipid peroxidation (LPO) accumulation. As shown in Fig. 4F, cells subjected to treatment with TCCHA upon laser irradiation (TCCHA + Laser) exhibited stronger green fluorescence signals than did those treated with TCCHA alone or Ce6 with laser irradiation, indicating that a higher level of LPO was induced by TCCHA nanoparticles upon laser irradiation. This suggests that PDT and the Fenton reaction have synergistic effects.

Therefore, we can conclude that TCCHA nanoparticles are capable of generating ⋅OH through a Cu-based Fenton-like reaction and generating ^1^O_2_ from Ce6 upon laser irradiation. Additionally, TCCHA nanoparticles alleviate hypoxia through Cu^2+^-mediated CAT activity and deplete GSH through Cu^2+^/TPH-mediated GPx activity. This bidirectional process ensures a cascade catalytic reaction that triggers ROS storms, which further leads to the accumulation of LPO and apoptotic cell death.

### In vivo biodistribution and antitumor efficacy

The in vivo biodistribution of TCCHA nanoparticles was investigated in HepG2 tumor-bearing BALB/c nude mice. First, a visualization study was conducted to investigate the tumor-targeting behavior of the TCCHA nanoparticles by using Ce6 as a fluorescence probe for in vivo near-infrared fluorescence imaging. As depicted in Fig. 5A, the Ce6 fluorescence in the tumor gradually increased over time and reached a stable level at 24 h postinjection. Consistent with the in vivo imaging results, ex vivo imaging (Fig. 5B) revealed the highest fluorescence intensity in the tumor tissue compared to that in the other major organs, confirming the remarkable tumor-targeting ability of the TCCHA nanoparticles. In addition, the pharmacokinetics of TCCHA nanoparticles were determined by monitoring Cu levels in the blood at different time points. The blood circulation half-life of TCCHA was preliminarily determined to be 5.27 h (Figure S12). The biodistribution of the TCCHA nanoparticles was further determined by detecting the Cu content in major organs/tumors via ICP analysis. The results showed that TCCHA nanoparticles were enriched at tumor sites and reached a maximum value of 10.1% ID g^− 1^ at 24 h postinjection (Fig. 5C), confirming the efficient accumulation and excellent targeting ability of TCCHA nanoparticles in tumors. TCCHA nanoparticles were also widely distributed in the liver and kidney, possibly due to reticuloendothelial system uptake. The biodistributions of Ce6 (Figure S13) and AL3818 (Figure S14) determined by HPLC analysis are similar to those of Cu^2+^. Both Ce6 and AL3818 had greater accumulation in tumors, reaching 13.4% and 11% ID/g at 24 h, respectively, which nearly matched the in vitro release behavior.

Thereafter, the antitumor effect of TCCHA nanoparticles was evaluated in HepG2 tumor-bearing mice. The therapeutic schedule is presented in Fig. 5D, and the mice were randomly divided into 6 groups. Digital photographs taken at specific time points are provided in Fig. 5E, which shows the changes in tumor volume during treatment. The resulting tumor growth curves are shown in Fig. 5F. During the observation period, the control group exhibited rapid tumor growth, while the Ce6 + Laser or AL3818 group exhibited relatively weak tumor inhibition. The TCCH + Laser or TCCHA group demonstrated enhanced antitumor efficacy. As expected, the TCCHA + Laser group exhibited the most significant tumor inhibition effect, wherein tumor growth was completely suppressed. After a 14-day treatment period, the tumors were removed, photographed, and weighed. The TCCHA + Laser treatment group had the smallest tumor volume (Fig. 5G) and the lowest tumor weight (0.114 g) (Fig. 5H). These findings further validate that TCCHA nanoparticles combined with laser irradiation can produce robust antitumor effects through the synergistic actions of CDT, PDT, and antiangiogenic therapy. Additionally, the in vivo biosafety of the aforementioned formulations was also assessed. There was no significant difference in body weight between the treatment groups and the control group throughout the entire treatment period (Fig. 5I), suggesting that TCCHA nanoparticles combined with laser irradiation pose no biosafety concerns in HepG2 tumor-bearing mice. Encouraged by the outstanding antitumor effects of TCCHA nanoparticles in HepG2 tumor-bearing mice, we further examined their antitumor efficacy in H22 tumor-bearing mice. The mice were photographed at predetermined time points, and representative digital images are displayed in Figure S15, which visually illustrates the changes in tumor volume throughout the treatment periods. Correspondingly, the tumor growth curves and mouse body weights are depicted in Figure S16 and Figure S17, respectively. As expected, the TCCHA + Laser group displayed the most significant tumor suppression effect. The final relative tumor volume in the TCCHA + Laser group was notably reduced to 0.6-fold, which was significantly smaller than that in the control group (11.8-fold), AL3818 (9.6-fold), Ce6 + Laser (6.7-fold), TCCH + Laser (3.4-fold), and TCCHA (3.3-fold) groups. The tumor microenvironment in vivo is complex in composition and contains multiple immune cell components including macrophages. Macrophage uptake capacity may have an impact on the anti-tumor efficacy of TCCHA, which deserves further in-depth study in the future.

Finally, we investigated the anticancer mechanism of the TCCHA nanoparticles at the histological level. As demonstrated in Figure S18, 5 mg/kg TCCHA in vivo effectively alleviated tumor hypoxia compared to that in the control group, which agreed with the in vitro results presented in Fig. 4D. This suggests that TCCHA nanoparticles are beneficial for enhancing the therapeutic effects of PDT. As shown in Fig. 5J, H&E staining of the cells in the TCCHA + Laser group revealed notable signs of nuclear pyknosis, cell membrane dissolution, and cell shrinkage, providing further evidence of the potent antitumor effect of the TCCHA nanoparticles. To gain a more comprehensive understanding of how TCCHA works to combat cancer, TUNEL (apoptosis indicator), Ki67 (proliferation indicator), and CD31 (angiogenesis indicator) staining of tumor tissues was carried out. Figure 5J shows that the TCCHA + Laser group had the strongest TUNEL fluorescence and the weakest Ki67 and CD31 fluorescence. In particular, the CD31 fluorescence intensity in the TCCHA and TCCHA + Laser groups was reduced compared with the AL3818 group, which proved that the introduction of AL3818 could fully overcome Cu^2+^-induced angiogenesis and further enhance the antitumor effect of TCCHA nanoparticles. Encouraged by these CD31 staining results, we assessed the anti-metastatic effect of TCCHA nanoparticles in a lung metastatic Hepa1-6 tumor model. The tumor-bearing mice were grouped and treated with PBS, AL3818, or TCCHA with or without laser irradiation. As indicated by the H&E-stained lung tissue images (Figure S19), TCCHA with or without laser irradiation had an efficient antimetastatic effect. Specifically, the average number of metastatic nodules per lung in the TCCHA + Laser group (180 ± 20) was nearly the same as TCCHA group (192 ± 14), and fewer than that in the control (251 ± 29) and AL3818 (231 ± 20) groups (Figure S20). Accordingly, the TCCHA + Laser and TCCHA groups had similar lung metastasis inhibition rates of 28% and 24%, respectively, which were much greater than that of the AL3818 group (8%) (Figure S21). All these findings suggest that TCCHA nanoparticles combined with laser irradiation can exert potent anticancer effects through a triple synergistic mechanism involving the induction of tumor cell apoptosis, the inhibition of tumor cell proliferation, and the suppression of tumor tissue angiogenesis.

## Conclusion

We successfully prepared a versatile copper-based nanoparticle, TCCHA, by a simple one-pot self-assembly strategy and revealed that TCCHA could act as a multienzyme for synergistic chemodynamic/photodynamic/antiangiogenic anticancer therapy. The TCCHA had a hydrodynamic size of 188 nm and a zeta potential of -20.1 mV and exhibited pH/GSH dual-responsive drug release behavior as well as TME-triggered enzyme catalytic activities, including Fenton-like, GPx-like, and CAT-like activities. TCCHA nanoparticles could be efficiently internalized by CD44 receptor-mediated cellular uptake, wherein TCCHA nanoparticles enhanced ⋅OH production, GSH depletion, and O_2_ generation, thus significantly enhancing the efficacy of CDT and PDT. Importantly, TCCHA nanoparticles demonstrated superior tumor suppression capability without obvious side effects and effectively inhibited tumor angiogenesis. In summary, this work offers an innovative approach for the construction of nanoparticles with multienzymatic activities, and the TCCHA platform provides a paradigm of catalysis-enhanced chemodynamic/photodynamic/antiangiogenic triple synergistic therapy against hepatocellular carcinoma.

### Electronic supplementary material

Below is the link to the electronic supplementary material.

Supplementary Material 1

## Data Availability

No datasets were generated or analysed during the current study.
