# Supplementary material for: Self-assembled copper-based nanoparticles for enzyme catalysis-enhanced chemodynamic/photodynamic/antiangiogenic tritherapy against hepatocellular carcinoma
Source: J Nanobiotechnology. 2024 Jun 26;22:375. doi: 10.1186/s12951-024-02626-x (PMC11202248; doi:10.1186/s12951-024-02626-x)
Supplement: Supplementary file 1 — Supplementary Material 1 [file 12951_2024_2626_MOESM1_ESM.docx]

**Self-assembled copper-based nanoparticles** **for enzyme catalysis-enhanced chemodynamic/photodynamic/antiangiogenic tritherapy against hepatocellular carcinoma**

Supplementary Material


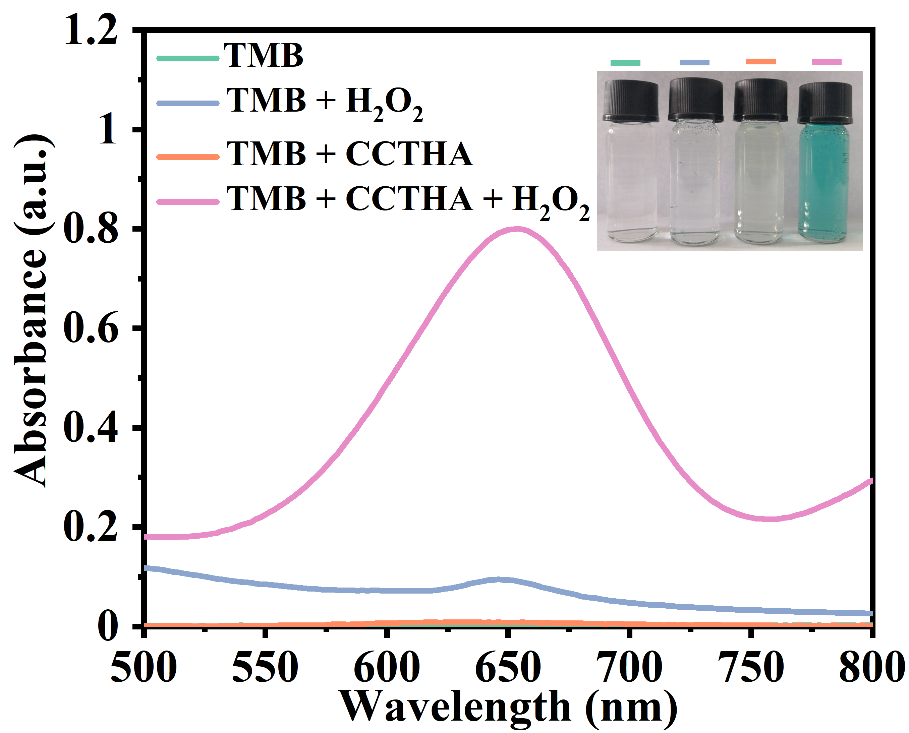


**Figure S1**. UV-vis spectra of TMB solutions after incubation with pristine H_2_O_2_, TCCHA and H_2_O_2_/TCCHA mixed solutions.


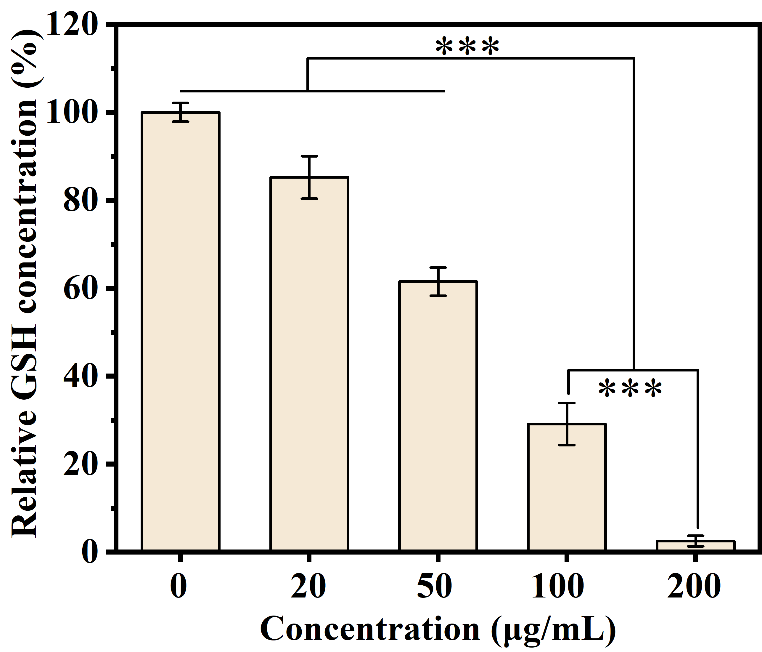


**Figure S2**. The relative GSH concentration after treatment with different concentrations of TCCHA. *n* = 3, *** *p* < 0.001.


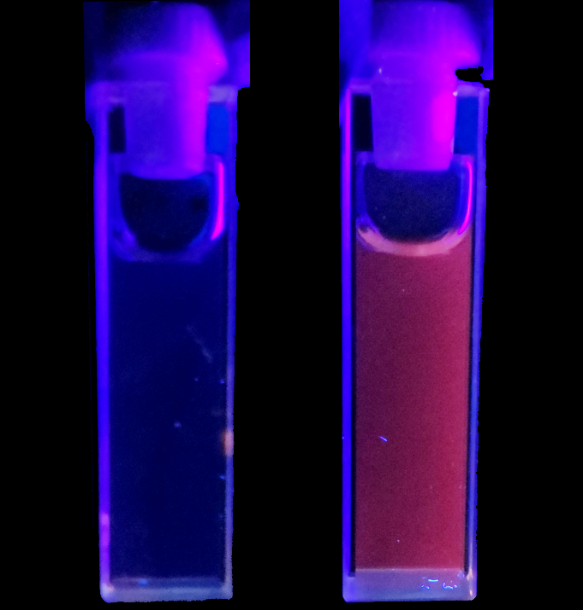


**Figure S3**. Photographs of TCCHA with or without 20 mM GSH incubation under UV light irradiation.


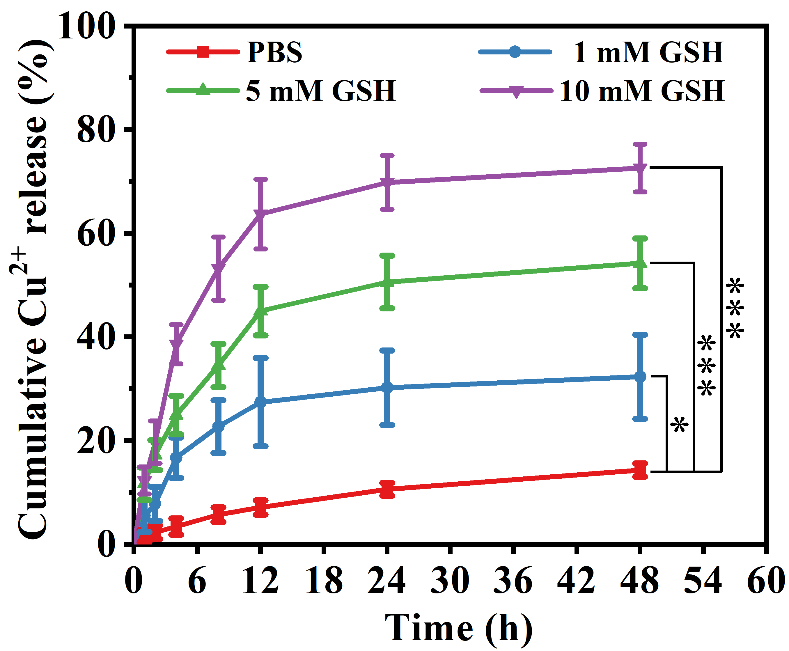


**Figure S4**. Cumulative release profiles of Cu^2+^ from TCCHA dispersed in in GSH solutions of varying concentrations. *n* = 3, * *p* < 0.05, *** *p* < 0.001.


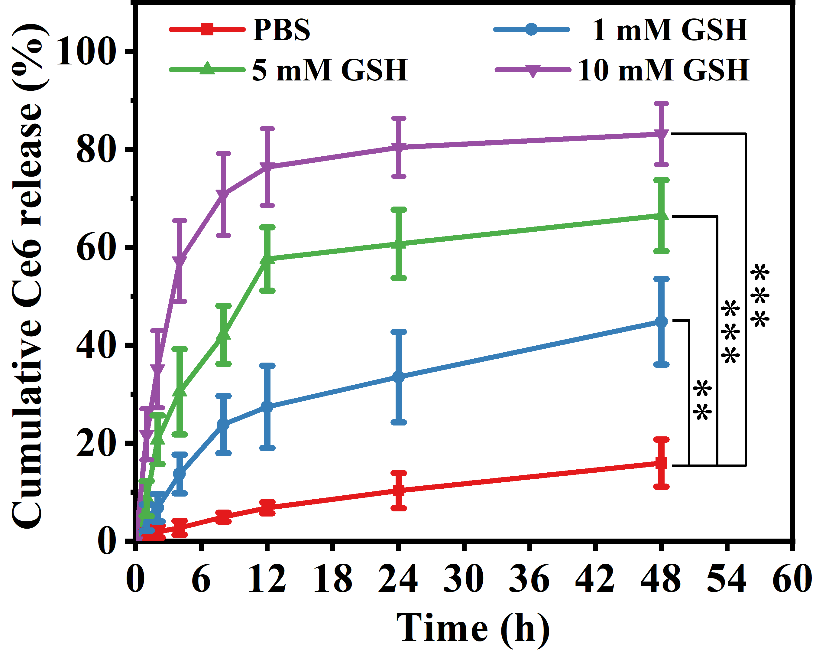


**Figure S5**. Cumulative release profiles of Ce6 from TCCHA dispersed in in GSH solutions of varying concentrations. *n* = 3, ** *p* < 0.01, *** *p* < 0.001.


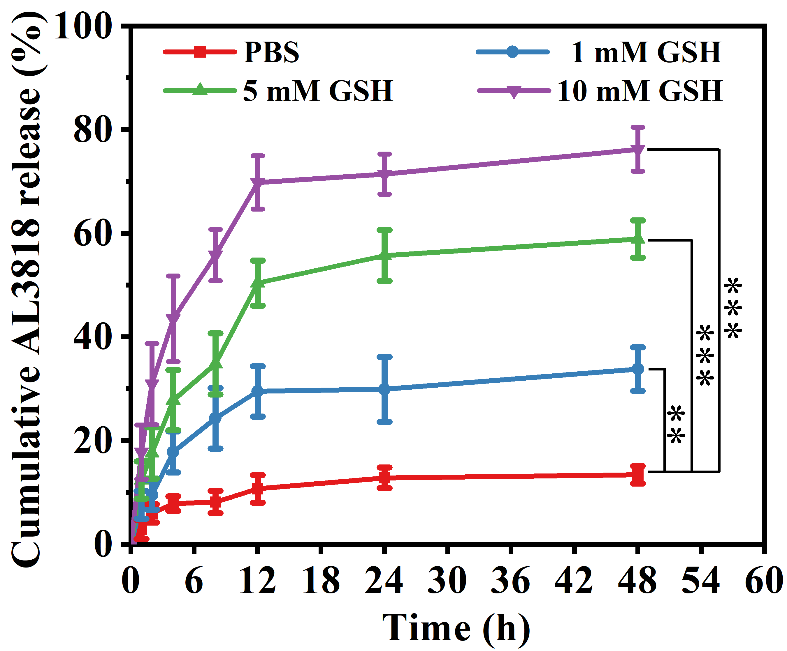


**Figure S6**. Cumulative release profiles of AL3818 from TCCHA dispersed in in GSH solutions of varying concentrations. *n* = 3, ** *p* < 0.01, *** *p* < 0.001.


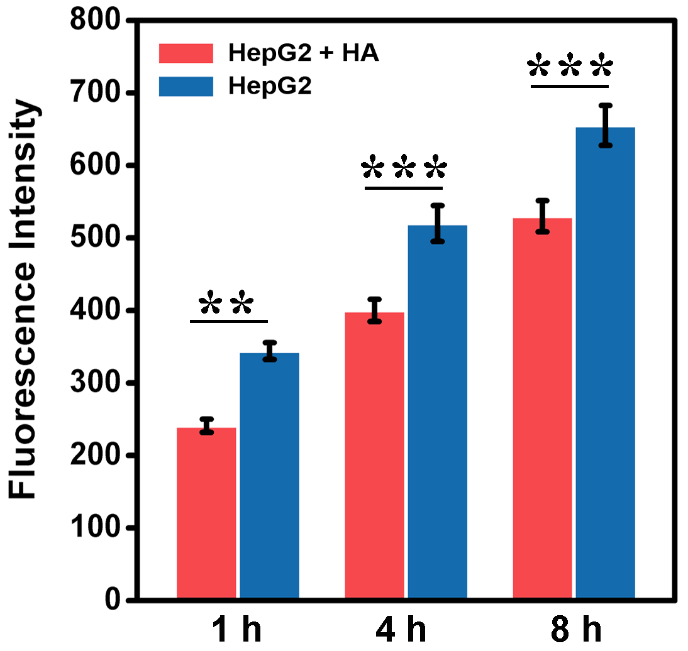


**Figure S7**. Quantitative analysis on the relative changes of intracellular Ce6 levels in HepG2 cells group or HA pretreatment HepG2 cells group (HepG2 + HA) for 1, 4, and 8 h. *n* = 3, ** *p* < 0.01, *** *p* < 0.001.


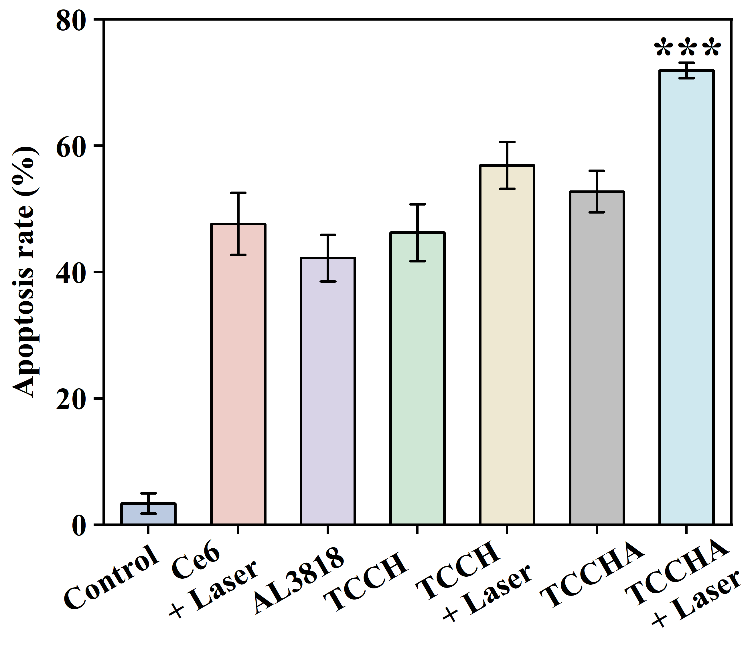


**Figure S8**. The results of the quantitative analysis of the apoptosis rates are shown in Figure 3H. *n* = 3, *** *p* < 0.001.





**Figure S9**. Quantitative analysis of H_2_O_2_ levels in HepG2 cells after treatment with various concentrations of TCCHA with or without H_2_O_2_ pretreatment. *n* = 3, ** *p* < 0.01.





**Figure S10**. Quantitative analysis of HIF-1α levels after various concentrations of TCCHA treatment with or without hypoxia pre-treatment. *n* = 3, ** *p* < 0.01.





**Figure S11**. The fluorescence intensity of DCF in Figure 4E


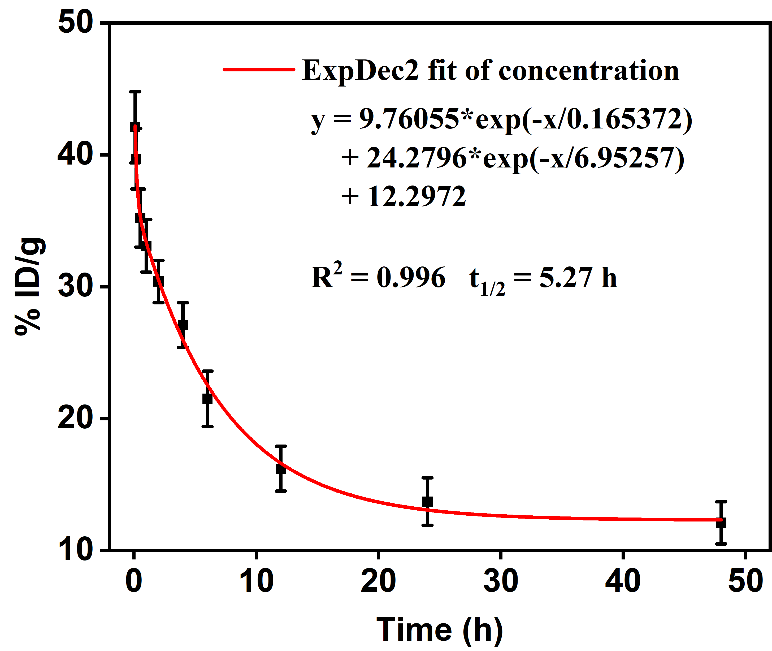


**Figure S12**. Pharmacokinetic profile of TCCHA. The data were determined by ICP-MS analysis of the Cu content in blood and are presented as a percentage of the injected dose (%ID).





**Figure S13**. Quantitative biodistribution of TCCHA in major organs and tumors at 24 h postinjection, as determined by HPLC analysis of Ce6 content and presented as a percentage of the injected dose (%ID).





**Figure S14**. Quantitative biodistribution of TCCHA in major organs and tumors at 24 h postinjection, as determined by HPLC analysis of AL3818 content and presented as a percentage of the injected dose (%ID).


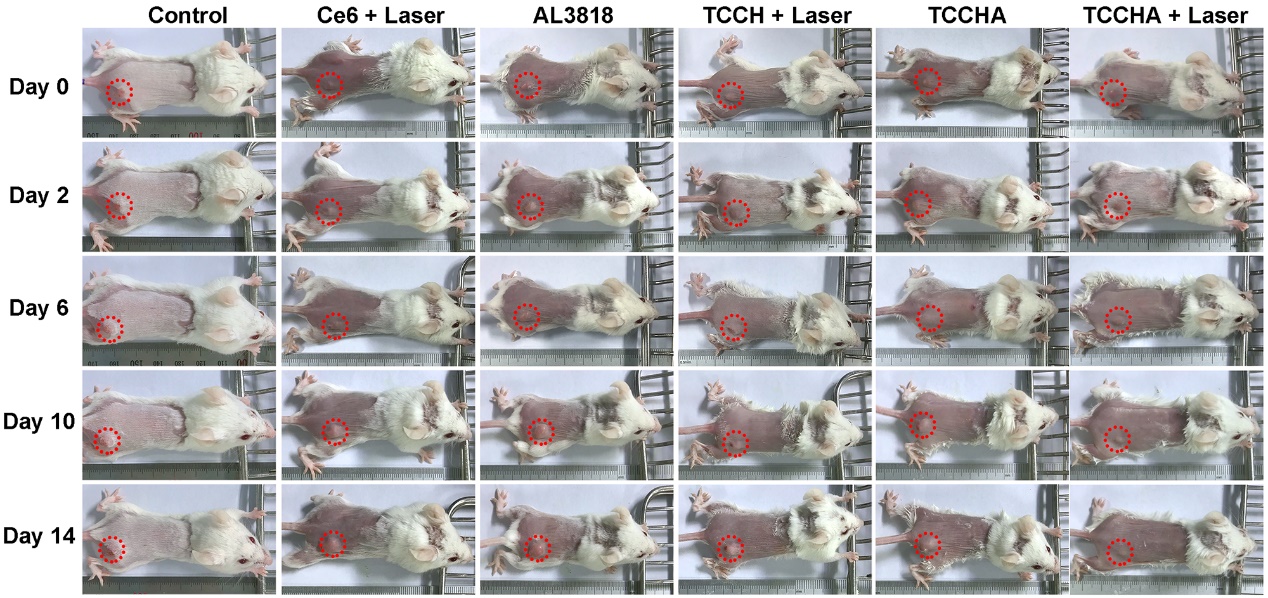


**Figure S15**. Digital photographs of mice taken at 0 d, 2 d, 6 d, 10 d and 14 d after treatment with different formulations of Ce6 + laser, AL3818, TCCH + laser, TCCHA, or TCCHA + laser. The tumor sites are marked with red circles.


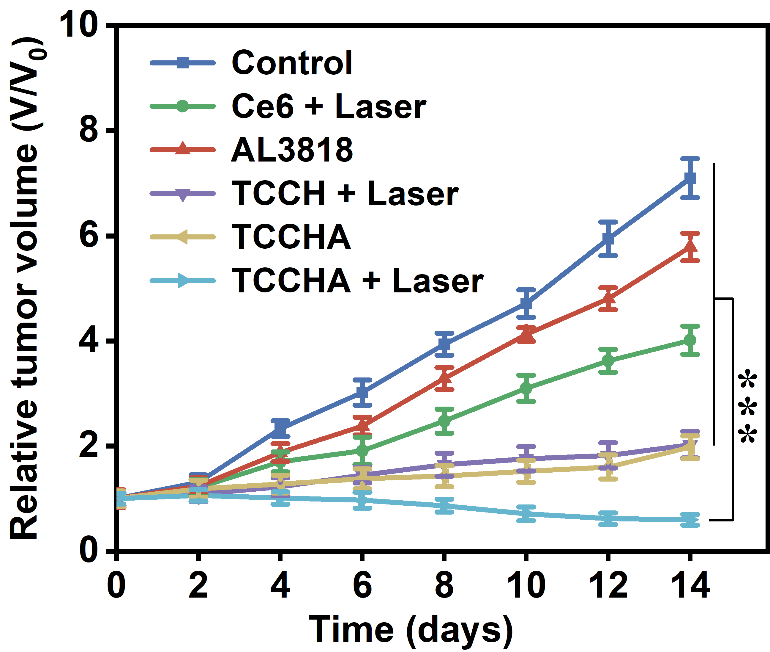


**Figure S16**. Tumor growth curves of H22 tumor-bearing mice in different treatment groups. *n* = 6, ****p* < 0.001.


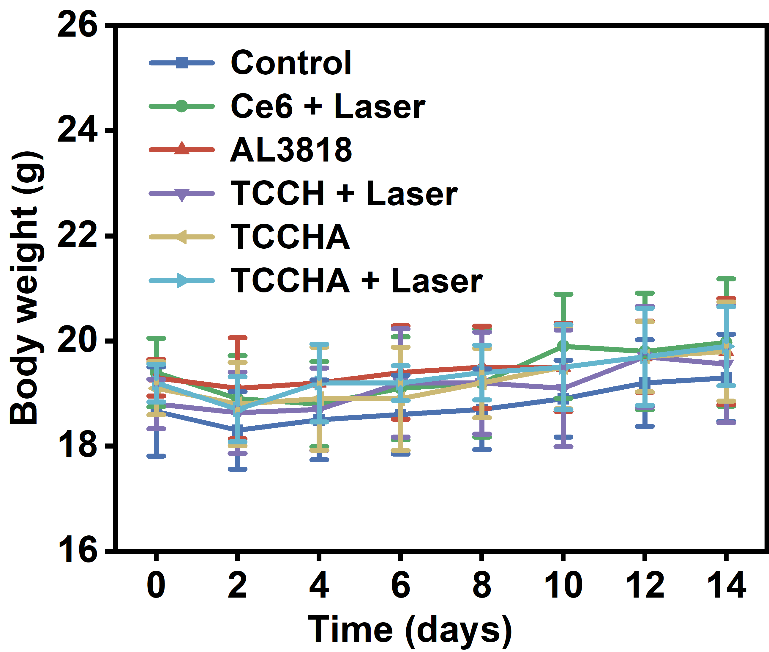


**Figure S17**. Body weight of H22 tumor-bearing mice received different treatments within 14 days.


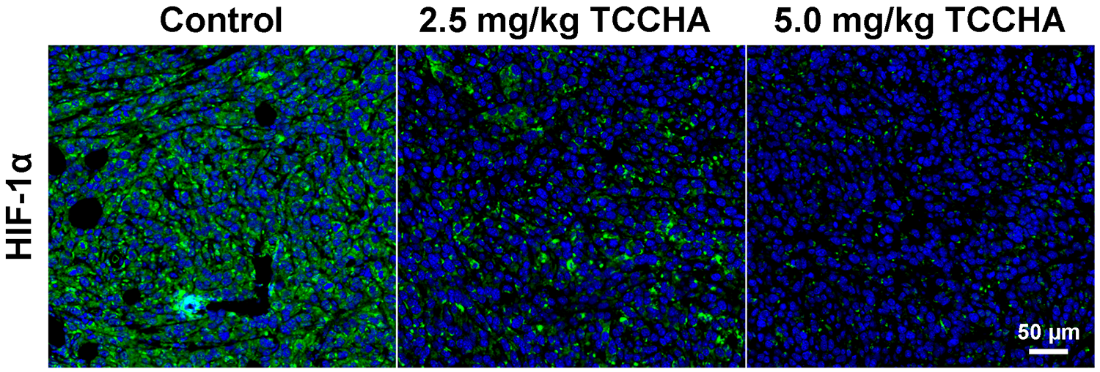


**Figure S18**. Immunofluorescence staining of HIF-1α in tumor sections after treatment with various concentrations of TCCHA for 14 days. (Blue: Hoechst-stained nuclei. Green: HIF-1α)


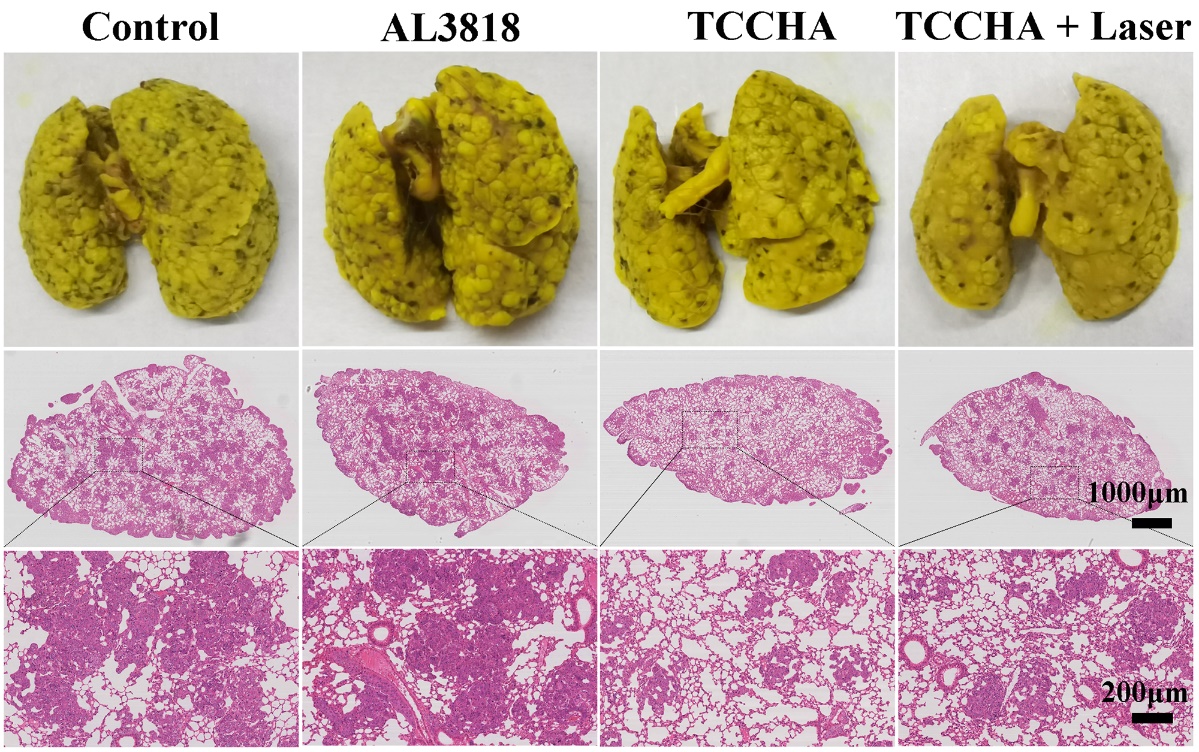


**Figure S19**. Representative photographs of lungs fixed by Bouin’s solution (upper panel) and H&E images of lung tissues in different groups. The black dotted squares denote metastatic nodules.


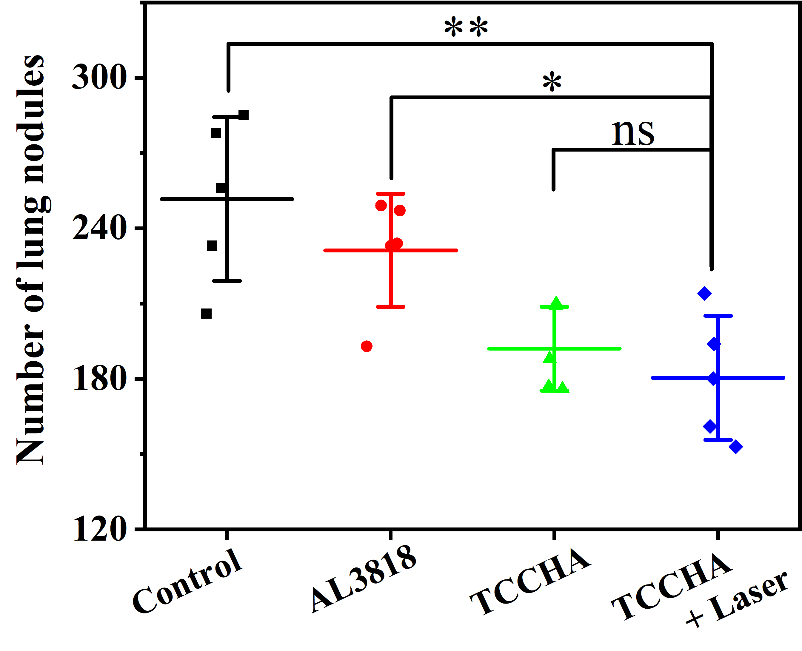


**Figure S20**. Numbers of lung metastatic nodules in Hepa1-6 lung metastatic mice in different treatment groups. *n* = 5, * *p* < 0.05, ** *p* < 0.01, “ns” represents no statistical significance.


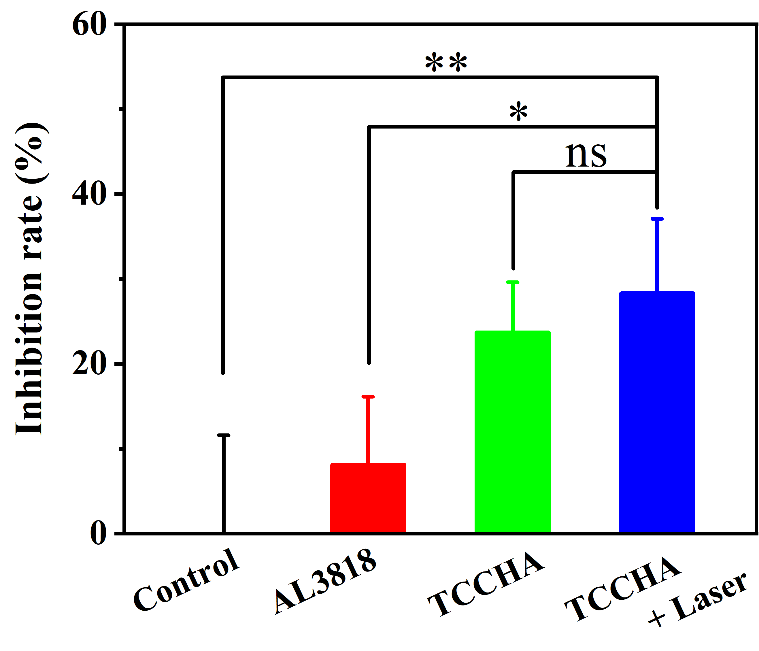


**Figure S21**. Relative lung metastasis inhibition rates in different treatment groups. *n* = 5, * *p* < 0.05, ** *p* < 0.01, “ns” represents no statistical significance.
